# String correction using the Damerau-Levenshtein distance

**DOI:** 10.1186/s12859-019-2819-0

**Published:** 2019-06-06

**Authors:** Chunchun Zhao, Sartaj Sahni

**Affiliations:** Department of Computer and Information Science and Engineering, University of Florida, Gainesville, 32611 FL USA

**Keywords:** Edit distance, Damerau-Levenshtein distance, Cache efficient, String correction

## Abstract

**Background:**

In the string correction problem, we are to transform one string into another using a set of prescribed edit operations. In string correction using the Damerau-Levenshtein (DL) distance, the permissible edit operations are: substitution, insertion, deletion and transposition. Several algorithms for string correction using the DL distance have been proposed. The fastest and most space efficient of these algorithms is due to Lowrance and Wagner. It computes the DL distance between strings of length *m* and *n*, respectively, in *O*(*mn*) time and *O*(*mn*) space. In this paper, we focus on the development of algorithms whose asymptotic space complexity is less and whose actual runtime and energy consumption are less than those of the algorithm of Lowrance and Wagner.

**Results:**

We develop space- and cache-efficient algorithms to compute the Damerau-Levenshtein (DL) distance between two strings as well as to find a sequence of edit operations of length equal to the DL distance. Our algorithms require *O*(*s* min{*m*,*n*}+*m*+*n*) space, where *s* is the size of the alphabet and *m* and *n* are, respectively, the lengths of the two strings. Previously known algorithms require *O*(*mn*) space. The space- and cache-efficient algorithms of this paper are demonstrated, experimentally, to be superior to earlier algorithms for the DL distance problem on time, space, and enery metrics using three different computational platforms.

**Conclusion:**

Our benchmarking shows that, our algorithms are able to handle much larger sequences than earlier algorithms due to the reduction in space requirements. On a single core, we are able to compute the DL distance and an optimal edit sequence faster than known algorithms by as much as 73.1% and 63.5%, respectively. Further, we reduce energy consumption by as much as 68.5%. Multicore versions of our algorithms achieve a speedup of 23.2 on 24 cores.

## Background

### Introduction

In the string correction problem, we are given two strings *A* and *B* and are required to find the minimum number of edit operations needed to transform *A* into *B*. The permitted edit operations are: (a) substitute a character in *A* to a different character, (b) insert a character into *A*, (c) delete a character of *A*, and (d) transpose two adjacent characters of *A*. When all four edit operations are permitted, the length of the optimal edit sequence is known as the Damerau-Levenshtein (DL) distance [1, 2]. Some applications limit the permissible edit operations to a subset of the stated four operations. As a result, string correction has been studied using other distance metrics as well. For example, the Levenshtein distance [1] is the length of the shortest sequence of substitutions, insertions, and deletions needed to transform *A* into *B*. This distance is used in the longest common subsequence problem [3], for example. When only substitutions are allowed, the length of the minimum edit sequence is the Hamming distance [4] and when only transpositions are allowed, this length is the Jaro distance [5].

The cost of an edit sequence may be generalized by using weights for the various operations. For example, in sequence alignment using the methods of Needleman and Wunsch [6] and Smith and Waterman [7], transpositions are not permitted, the cost of a substitution depends on the two characters involved, and there is a gap penalty. The string-to-string correction algorithm of Lowrance and Wagner [8] uses a cost of *S* for a substitution, *I* for an insertion, *D* for a deletion, and *T* for a transposition and requires 2*T*≥*I*+*D*. We note that the costs used in computing the DL distance are *S*=*I*=*D*=*T*=1 and that these costs satisfy the 2*T*≥*I*+*D* requirement of the algorithm of Lowrance and Wagner [8]. In fact, the best algorithm currently known for the DL distance is the one in [8] with edit operation costs set to 1.

Spelling error correction [9–11], data clustering and data mining [12], comparing packet traces [13], quantifying the similarity of DNA/RNA/protein sequences, gene finding, and gene function prediction [14] are some of the applications of the DL distance. While, in spelling error correction, the strings *A* and *B* are relatively short, in other applications, these strings may be quite long. For example, the length of a protein sequence may exceed 300,000 [15].

Bard [10] has shown that the DL distance is a true metric; that is, it satisfies 1) non-negativity, 2) identity, 3) symmetry, and 4) triangle inequality. The algorithm of Bard [10] computes the DL distance in *O*(*mn*∗ max{*m*,*n*}) time, where *m* is the length of string *A* and *n* is the length of *B*. This algorithm uses *O*(*mn*) space. Hyyro [16] has developed a bit-parallel algorithm to determine whether the DL distance between two strings is less than a specified threshold. This bit-parallel algorithm was tested using DNA sequences of length up to 10,000.

In an effort to reduce time complexity, Oommen and Loke [17] consider restricting edit sequences so that no substring is edited more than once. We illustrate this restriction using the example given in [18]. The string CA may be transformed into ABC using the edit sequence CA (transposition) → AC (insertion) → ABC. So, the DL distance between CA and ABC is 2. With the restriction of [17], the second operation in this edit sequence is not permitted as it involves re-editing AC, which resulted from the first edit operation. The restricted DL distance is 3, which corresponds to the restricted edit sequence CA (deletion) → A (insertion) → AB (insertion) → ABC. The restricted DL distance is not a metric as it does not satisfy the triangle inequality.

The algorithm of Lowrance and Wagner [8] computes the DL distance in *O*(*mn*) time while also using *O*(*mn*) space. This is the fastest and most space efficient algorithm known for string correction using the DL distance.

Neither the algorithm of Bard [10] nor that of Lowrance and Wagner [8] is practical when *m* and *n* are large due to their excessive space requirement. The former algorithm becomes impractical also due to its excessive run time. In this paper, we focus on the development of algorithms that are more space, time, and energy efficient than that of Lowrance and Wagner [8]. To obtain space efficiency, we observe that the DL distance can be computed by retaining only *O*(*sm*) or *O*(*sn*) data, where *s* is the size of the alphabet. We note that, when *m* and *n* are large, *s* is much smaller than *m* and *n*. In fact, *s*=4 for RNA and DNA sequences and *s*=20 for protein sequences and the length of these sequences is often orders of magnitude larger than *s*.

### Cache model

To analyze the cache performance of our algorithms, we use the rather simple cache model which has been used by us successfully in our past work [19]. In this model we have a single-level cache that has *l* cache lines of size *w*, where *w* is the number of data items that can be stored in one cache line. So, when the data size is 4 bytes and *w*=8, each cache line is 32 bytes. The size (i.e., capacity) of our one-level cache is *lw*. In accordance with this cache model, we assume that main memory is divided into blocks whose size is the same as that of a cache line (i.e., *w* words each). When we attempt to read a piece of data that is not in the cache, a read miss occurs. A read miss causes the corresponding block of main memory to be read into a cache line. When the cache is full, this read miss requires us to first evict the block that is in the least recently used (LRU) cache line. This eviction results in a write of the evicted block to main memory in case the evicted block has changed. A write miss occurs when we attempt to write data that is not in a cache line. At this time, the corresponding block of main memory is read into a cache line and the data we wish to write is written to this cache line.

Notice that every read and write miss results in a read access of main memory; some read and write misses also result in the writing of a cache line to main memory.

Today’s computers actually employ multiple levels of cache and a far more sophisticated and proprietary cache servicing policy combined with prefetching to hide memory latency. As a result, it is extremely difficult to analyze cache performance using a realistic cache model. The described simple cache model is amenable to analysis and our experiments establish its usefulness for this purpose as algorithms with reduced cache misses using this model actually run faster on computers with more sophisticated cache architectures, replacement policies, and prefetching techniques.

### Classical DL distance algorithm

Wagner and Fischer [20] developed the notion of a *trace*, which is useful in reasoning about edit sequences that are limited to substitutions, insertions, and deletions. Lowrance and Wagner [8] extended this notion to include the transposition operation. A *trace* for the strings *A*=*a*_1_⋯*a*_*m*_ and *B*=*b*_1_⋯*b*_*n*_ is a set *T* of lines, where the endpoints *u* and *v* of a line (*u*,*v*) denote positions in *A* and *B*, respectively. A set of lines *T* is a trace iff:

1. For every (*u*,*v*)∈*T*, *u*≤*m* and *v*≤*n*.
2. The lines in *T* have distinct *A* positions and distinct *B* positions. That is, no two lines in *T* have the same *u* or the same *v*.

**Fig. 1:**
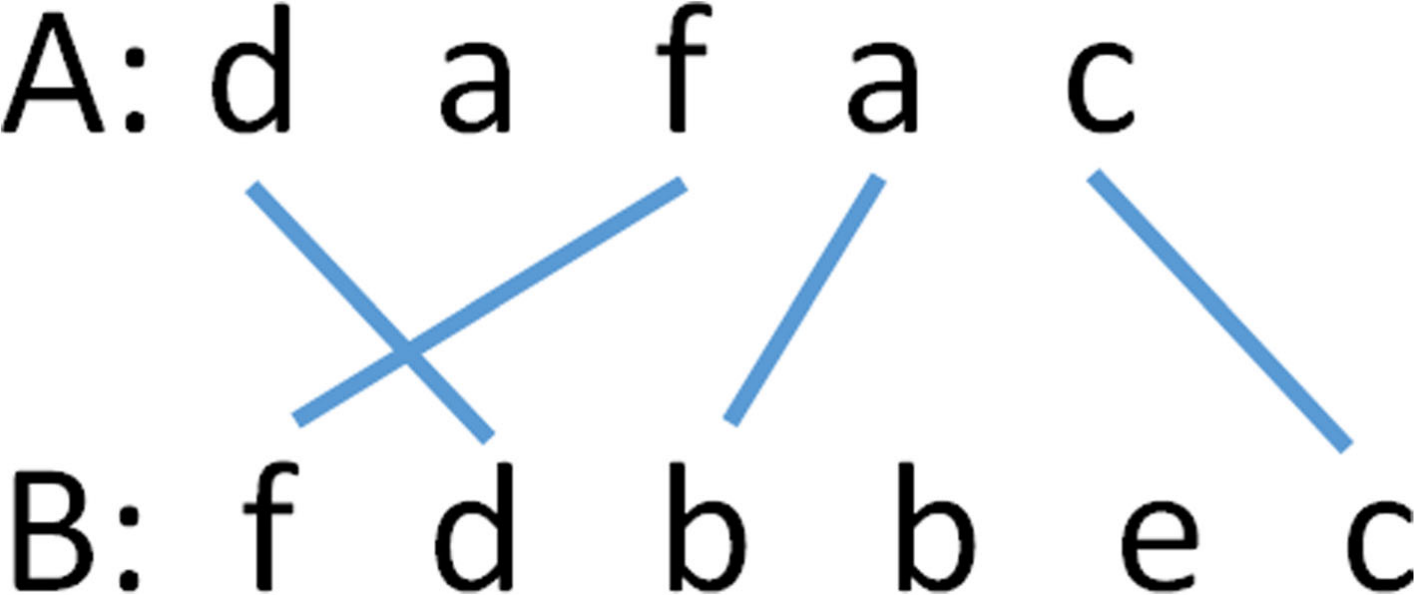
DL trace example

A line (*u*,*v*) is *balanced* iff *a*_*u*_=*b*_*v*_ and two lines (*u*_1_,*v*_1_) and (*u*_2_,*v*_2_) cross iff (*u*_1_<*u*_2_) and (*v*_1_>*v*_2_). As an example, consider *A*=*dafac* and *B*=*fdbbec*. The set of lines *T*={(1,2),(3,1),(4,3),(5,6)} satisfies the requirements for a trace. Line (4,3) is not balanced as *a*_4_≠*b*_3_. The remaining 3 lines in the trace are balanced. The lines (1,2) and (3,1) cross. This trace may be depicted as a diagram as in Fig. 1.

In a trace, an unbalanced line denotes a substitution operation and a balanced line denotes retaining the character of *A*. If *a*_*i*_ has no line attached to it, *a*_*i*_ is to be deleted and when *b*_*j*_ has no attached line, it is to be inserted. When two balanced lines (*u*_1_,*v*_1_) and (*u*_2_,*v*_2_) cross, $a_{u_{1}+1} \cdots a_{u_{2}-1}$ are to be deleted from *A* making $a_{u_{1}}$ and $a_{u_{2}}$ adjacent, then $a_{u_{1}}$ and $a_{u_{2}}$ are to be transposed, and finally, $b_{v_{2}+1} \cdots b_{v_{1}-1}$ are to be inserted between the just transposed characters of *A*.

The edit sequence corresponding to the trace of Fig. 1 is delete *a*_2_, transpose *a*_1_ and *a*_3_, substitute *b* for *a*_4_, insert *b*_4_=*b* and *b*_5_=*e*, retain *a*_5_. The cost of this edit sequence is 5.

Lowrance and Wagner [8] have proved the following properties:

- The cost of a trace equals the number of unbalanced lines plus the number of positions in *A* and *B* not touched by a line plus the number of line crossings.
- There is a trace whose cost equals that of an optimal edit sequence (Theorem 2 of [8]). Since every trace corresponds to an edit sequence, it follows that the edit sequence that corresponds to a minimum cost trace is optimal.
- There is a minimum cost trace in which each line crosses at most one other line and in which every line that crosses another is balanced (Theorem 4 of [8]).
- There is trace *T* that satisfies property P3 and for every pair of crossing lines (*u*_1_,*v*_1_), (*u*_2_,*v*_2_), *u*_1_<*u*_2_ in *T*, (a) $a_{i} \neq a_{u_{1}} = b_{v_{1}}\phantom {\dot {i}\!}$, *u*_1_<*i*<*u*_2_ and (b) $b_{j} \neq b_{v_{2}} = a_{u_{2}}\phantom {\dot {i}\!}$, *v*_2_<*j*<*v*_1_. In words, *u*_1_ is the last (i.e., rightmost) occurrence of $b_{v_{1}}$ in *A* that precedes position *u*_2_ of *A* and *v*_2_ is the last occurrence of $a_{u_{2}}$ in *B* that precedes position *v*_1_ of *B*. We refer to these positions as $lastA[\!u_{2}][\!b_{v_{1}}]\phantom {\dot {i}\!}$ and $\phantom {\dot {i}\!}lastB[\!v_{1}][\!a_{u_{2}}]$, respectively (Theorem 5 of [8]).

Let *H*_*ij*_ be the DL distance between *A*[ 1:*i*] to *B*[ 1:*j*]. So, *H*_*mn*_ is the DL distance between *A* and *B*. The following dynamic programming recurrence follows from properties P1-P4 of a trace.

1 $$ H_{i,0} = i,\ H_{0,j} = j, \ 0 \le i \le m, \ 0 \le j \le n  $$

When *i*>0 and *j*>0,

2 $$ {}H_{i,j} \,=\, \min\left\{\! \begin{array} {lcr} H_{i-1,j-1}+ c(a_{i},b_{j}) \\ H_{i,j-1}+ 1 \\ H_{i-1,j}+ 1 \\ H_{k-1,l-1} + (i-k-1)+ 1 + (j-l-1) \\ \end{array} \right.   $$

where *c*(*a*_*i*_,*b*_*j*_) is 1 if *a*_*i*_≠*b*_*j*_ and 0 otherwise, *k*=*lastA*[ *i*][ *b*_*j*_] and *l*=*lastB*[ *j*][ *a*_*i*_]. If *k* or *l* do not exist, then case 4 of the recurrence does not apply.

**Fig. 2:**
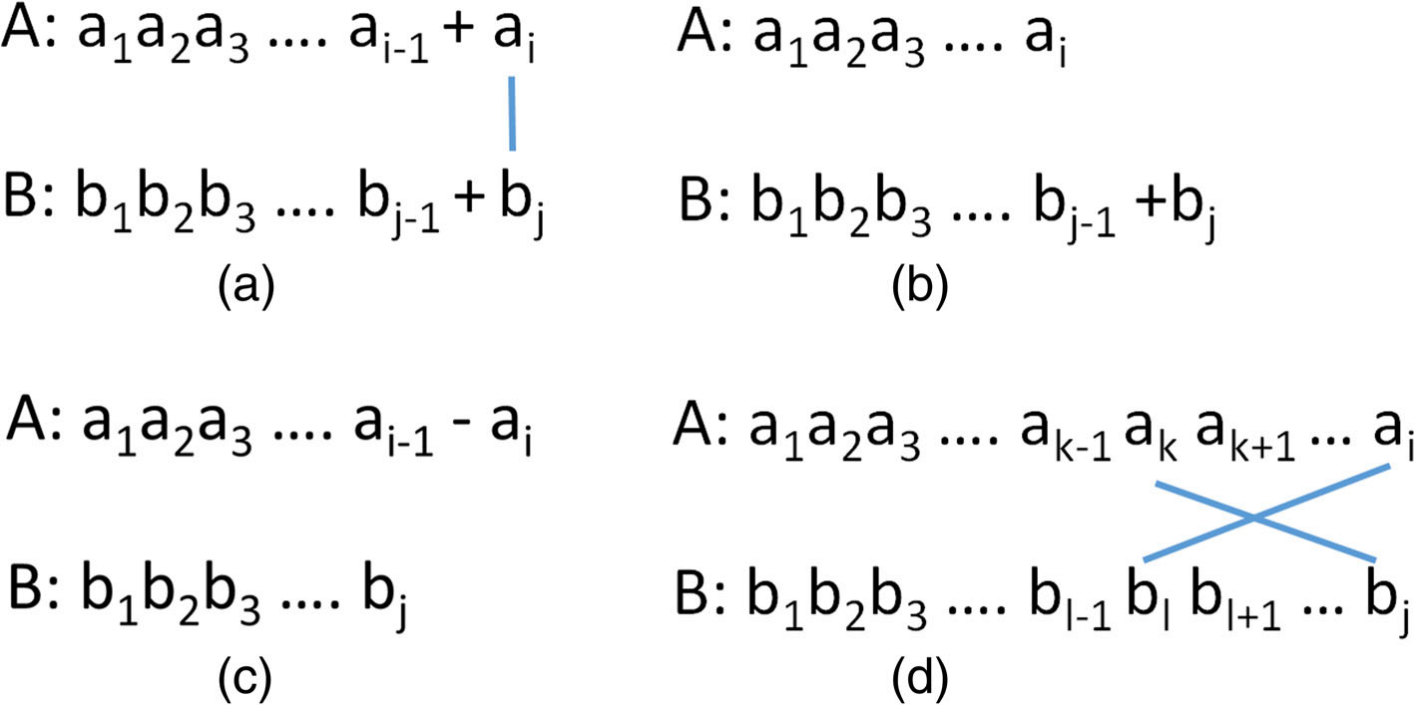
DL trace recurrence. **a** substitution **b** insertion **c** deletion **d** translate A[k:i] to B[l:j] where (a _k_,b _j_) and (b _l_,a _i_) form a transposition opportunity

Figure 2 illustrates the four cases of this recurrence. These cases correspond to the four possibilities for an optimal trace that transforms *A*[ 1:*i*] into *B*[ 1:*j*] and satisfies properties P2-P4. Such a trace may (a) contain the line (*i*,*j*), (b) contain no line that touches *b*_*j*_, (c) contain no line that touches *a*_*i*_, or (d) have crossing balanced lines that involve *a*_*i*_ and *b*_*j*_. Figure 2a illustrates the first case, which is a substitution between *a*_*i*_ and *b*_*j*_; we optimally transform *A*[ 1:*i*−1] into *B*[ 1:*j*−1] and then substitute *b*_*j*_ for *a*_*i*_. If *a*_*i*_=*b*_*j*_, the substitution cost is 0, otherwise it is 1. Figure 2b shows the second case. Here, *b*_*j*_ is inserted at the end of *B*[ 1:*j*−1] following an optimal transformation of *A*[ 1:*i*] into *B*[ 1:*j*−1]. Figure 2c shows the third case in which *a*_*i*_ is deleted from *A*[1:*i*] following an optimal transformation of *A*[ 1:*i*−1] into *B*[ 1:*j*]. Figure 2d shows the case of crossing balanced lines (*i*,*l*) and (*k*,*j*). Here, *A*[ 1:*k*−1] must be optimally transformed into *B*[ 1:*l*−1]. Note that to perform the crossing operation, we must delete *i*−*k*−1 characters from *A*, do an adjacent character transposition in *A*, and then insert *j*−*l*−1 characters from *B* between the two just transposed positions. So, the cost is (*i*−*k*−1)+1+(*j*−*l*−1).

Algorithm 1 is the pseudocode to compute *H* using Eqs. 1 and 2. This is a simplification of the pseudocode given in Lowrance and Wagner [8] to the case when each edit operation has unit cost. In this algorithm, *last*_*row*_*id*[*c*] keeps track of the last occurrence of character *c* in *A* (note that this is a row index of *H*) and *last*_*col*_*id* keeps track of the last occurrence of *a*_*i*_ in *B*.


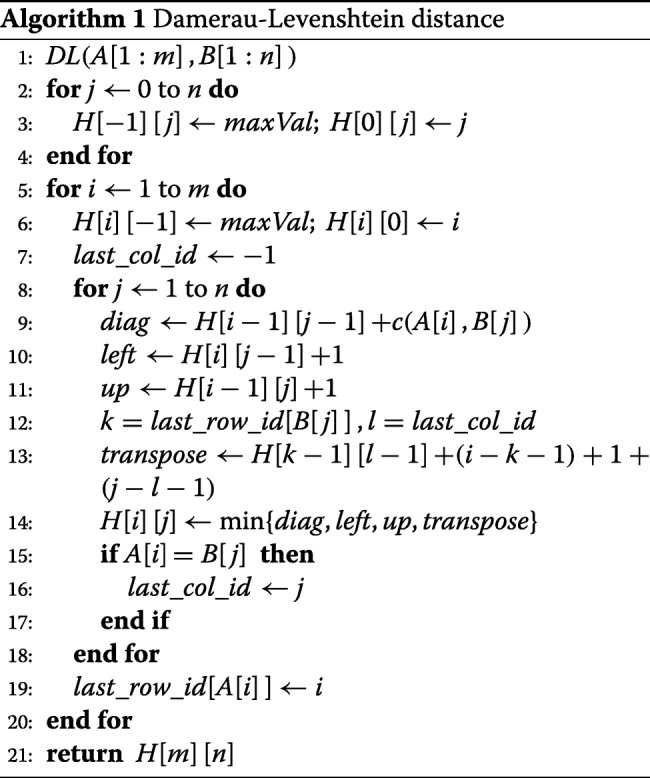


We shall refer to Algorithm 1 as algorithm *DL*. Its time and space complexities are readily seen to be *O*(*mn*). Once *H* has been computed using algorithm *DL*, an optimal trace may be obtained in *O*(*m*+*n*) additional time using a standard dynamic programming traceback. We refer to the combination of *DL* and the traceback as algorithm *DL*_*TRACE*.

The total number of cache misses is dominated by the read and write misses of the array *H*. So, we count only these misses. In each iteration of the loop for computing row *i* of *H*, we need the elements of rows *i* and *i*−1 of *H* in left-to-right order as in Algorithm 1 lines 9-11 and 14. Since these rows are read from main memory in blocks of size *w* and row *i* is written to main memory in blocks of this size, lines 9-11 and 14 result in 2*n*/*w* read accesses and *n*/*w* write accesses for each *i*. These lines, therefore, result in 3*mn*/*w* cache misses over the entire execution of *DL*. Line 13 makes one read access of *H* per iteration and so contributes at most *mn* to the total cache-miss count. Hence, the cache-miss count for algorithm *DL* is approximately *mn*(1+3/*w*).

## Methods

### Single-core algorithms

In this section, we develop four linear-space single-core algorithms for string correction using the DL distance. All four run in *O*(*mn*) time. The first two (*LS*_*DL* and *Strip*_*DL*) compute only the score *H*_*mn*_ of the optimal trace; they differ in their cache efficiency. The last two (*LSDL*_*TRACE* and *Strip*_*TRACE*) compute an optimal trace.


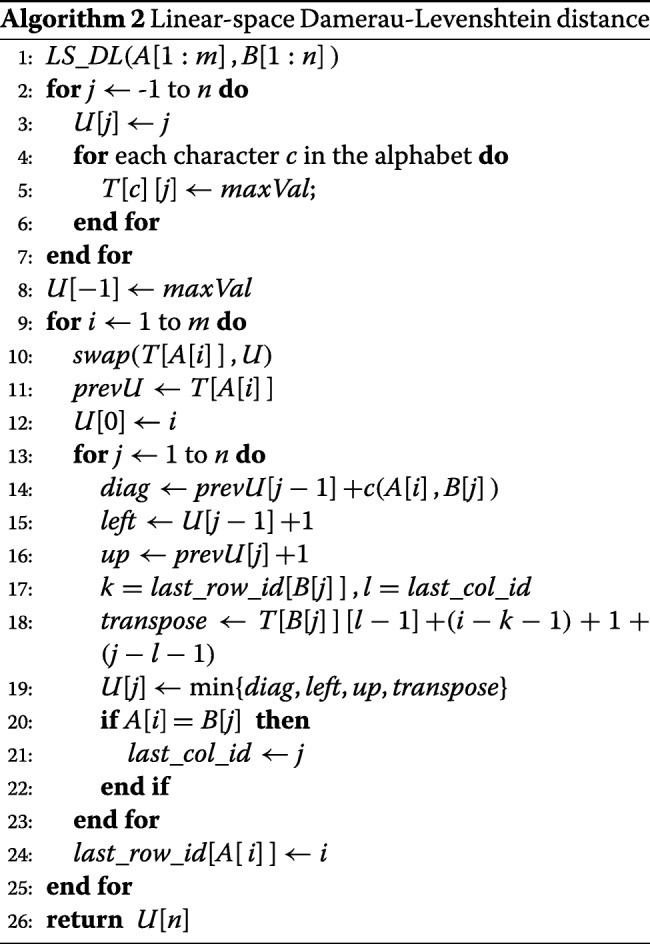


#### The linear space algorithm *LS*_*DL*

Let *s* be the size of the alphabet. Instead of using the array *H* used in *DL*, algorithm *LS*_*DL* uses a one-dimensional array *U*[ −1:*n*] and a two-dimensional array *T*[ 1:*s*][ −1:*n*]. These two arrays have a space requirement of *O*((*s*+1)*n*) = *O*(*n*) for constant *s*. When *m*<*n*, one may swap *A* and *B* to reduce the required memory. Adding the memory needed for *A* and *B*, the space complexity is *O*(*s* min{*m*,*n*}+*m*+*n*) = *O*(*m*+*n*) when *s* is a constant.

As in algorithm *DL*, the *H*_*ij*_ values are computed by rows. The one-dimensional array *U* is used to save the *H*[ *i*][ ∗] values computed by algorithm *DL* when row *i* is being computed. Let *H*[ *w*][ ∗] be the last row computed for character *c*. Then, *T*[ *c*][ ∗] is row *w*−1 of *H*. Algorithm 2 gives the pseudocode for *LS*_*DL*. Its correctness follows from the correctness of algorithm *DL*. Note that *swap*(*T*[*A*[ *i*]],*U*) takes *O*(1) time as pointers to 2 one-dimensional arrays are swapped rather than the content of these arrays. The cache-miss count for *LS*_*DL* is the same as that for *DL* when *n* is suitably large as both have the same data access pattern. However, for smaller instances *LS*_*DL* will exhibit much better cache behavior. For example, because of its use of much less memory, we may have enough LLC cache to store all the data in *LS*_*DL* but not in *DL* (*O*(*sn*) vs *O*(*mn*)).

#### The cache-efficient linear-space algorithm *Strip*_*DL*

**Fig. 3:**
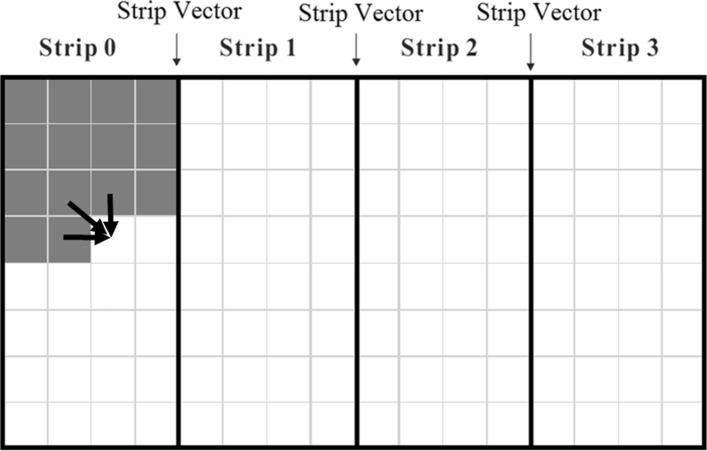
Computing *H* by strips

When (*s*+1)*n* is larger than the size of the LLC cache, we may reduce cache misses relative to algorithm *LS*_*DL* by computing *H*_*ij*_ by strips of width *q*, for some *q* less than *n* (the last strip may have a width smaller than *q*). This is shown in Fig. 3. The strips are computed in the order 0, 1,... using algorithm *LS*_*DL*. However, the space needed by *T* and *U* in *LS*_*DL* is reduced to (*s*+1)*q* as the strip width is *q* rather than *n*. By choosing *q* small enough, we can ensure that blocks of the *T* and *U* arrays used by *LS*_*DL* are not evicted from cache once they are brought in. So, if each entry of *T* and *U* takes 1 word, then when the cache size is *lw*, we have *q*<*lw*/(*s*+1). Note that, in addition to *T* and *U*, the cache needs to hold partials of *A*, *B* and other arrays needed to pass the data from one strip to the next.

To pass the data from one strip to next, we use an additional one-dimensional array *strip* of size *m* and a two-dimensional *s*∗*m* array *V*. The array *strip* records the values of *H* computed for the rightmost column in the strip. *V*[ *c*][*i*] gives the *H* value in the rightmost column *j* of row *i* of *H* that is (a) in a strip to the left of the one currently being computed and (b) *c*=*B*[ *j*].

The pseudocode for *Strip*_*DL* is given in Algorithm 3. For clarity, this pseudocode uses two *strip* arrays (lines 18 and 30) and two *V* arrays (lines 24 and 32). One set of arrays is used to fetch data calculated for the previous strip and the other set for data that is to be passed to the next strip. In the actual implementation, we use a single *strip* array and a single *V* array overwriting values received from the previous strip with values to be passed to the next strip.


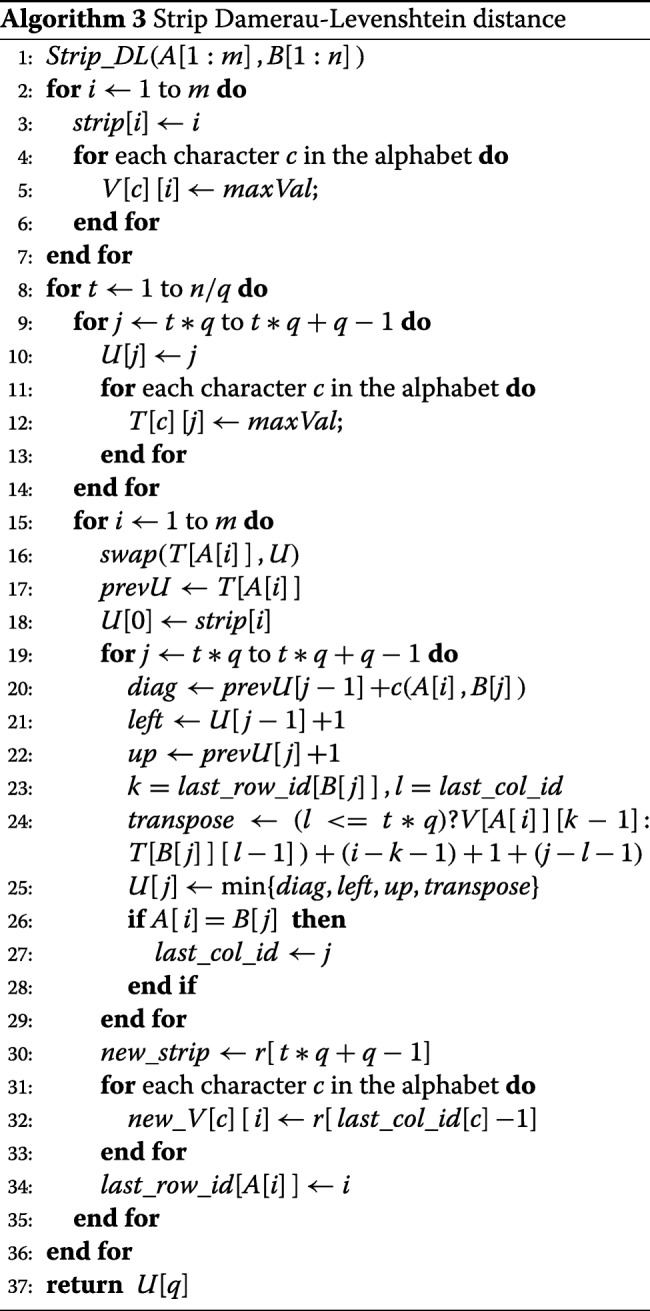


The time and complexity of *Strip*_*DL* are, respectively, *O*(*mn*) and *O*((*s*+1)*m*+(*s*+1)*q*+*n*) = *O*(*sm*+*sq*+*n*) = *O*(*sm*+*n*) as *q* is a constant. When *m*>*n*, we may switch *A* and *B* to conserve memory and so the space complexity becomes *O*(*s* min{*m*,*n*}+*m*+*n*) = *O*(*m*+*n*) for constant *s*.

When we analyze the cache miss, we note that *q* is chosen such that *U* and *T* fit into cache. We make the reasonable assumption that the LRU replacement rule does not cause any block of *U* or *T* to be evicted during the running of algorithm *Strip*_*DL*. As a result, the total number of cache misses due to *U* and *T* is independent of *m* and *n* and so may be ignored in the analysis. The initialization of *strip* and *V* results in *m*/*w* and (*s*+1)*m*/*w* read accesses, respectively. The number of write accesses is approximately the same as the number of read accesses. The computation for each strip accesses the array *strip* in ascending order of index. This results in (approximately) the same number of cache misses as made during the initialization phase. Hence, the total number of cache misses due to *strip* is approximately (2*m*/*w*)(*n*/*q*+1). For *V*, we note that when computing the current strip, the elements in any row of *V* are accessed in non-decreasing order of index (i.e., from left to right) and that we need to retain, in cache, only the most recently read value for each character of the alphabet (i.e., at most *s* values are to be retained). Making the assumption that a *V* value is evicted from cache only when a new value for the same character is accessed, the total number of read misses from *V* when computing a single strip is *sm*/*w*. The number of write misses is approximately the same. So, *V* contributes (2*sm*/*w*)(*n*/*q*+1). Hence, the total number of cache misses for algorithm *Strip*_*DL* is ≈2(*s*+1)*mn*/(*wq*) when *m* and *n* are large.

Recall that the approximate cache-miss count for algorithms *DL* and *LS*_*DL* is *mn*(1+3/*w*). This is (*wq*+3*q*)/(2*s*+2) times that for *Strip*_*DL*.

#### The linear-space trace algorithm *LSDL*_*TRACE*

Although algorithms *LS*_*DL* and *Strip*_*DL* determine the score (cost) of an optimal trace (and hence of an optimal edit sequence) that transforms *A* into *B*, these algorithms do not save enough information to actually determine an optimal trace. To determine an optimal trace using linear space, we adopt a divide-and-conquer strategy similar to that used by Hirschberg [21] for the simple string editing problem (i.e., transpositions are not permitted) and Myers and Miller [22] for the sequence alignment problem.

**Fig. 4:**
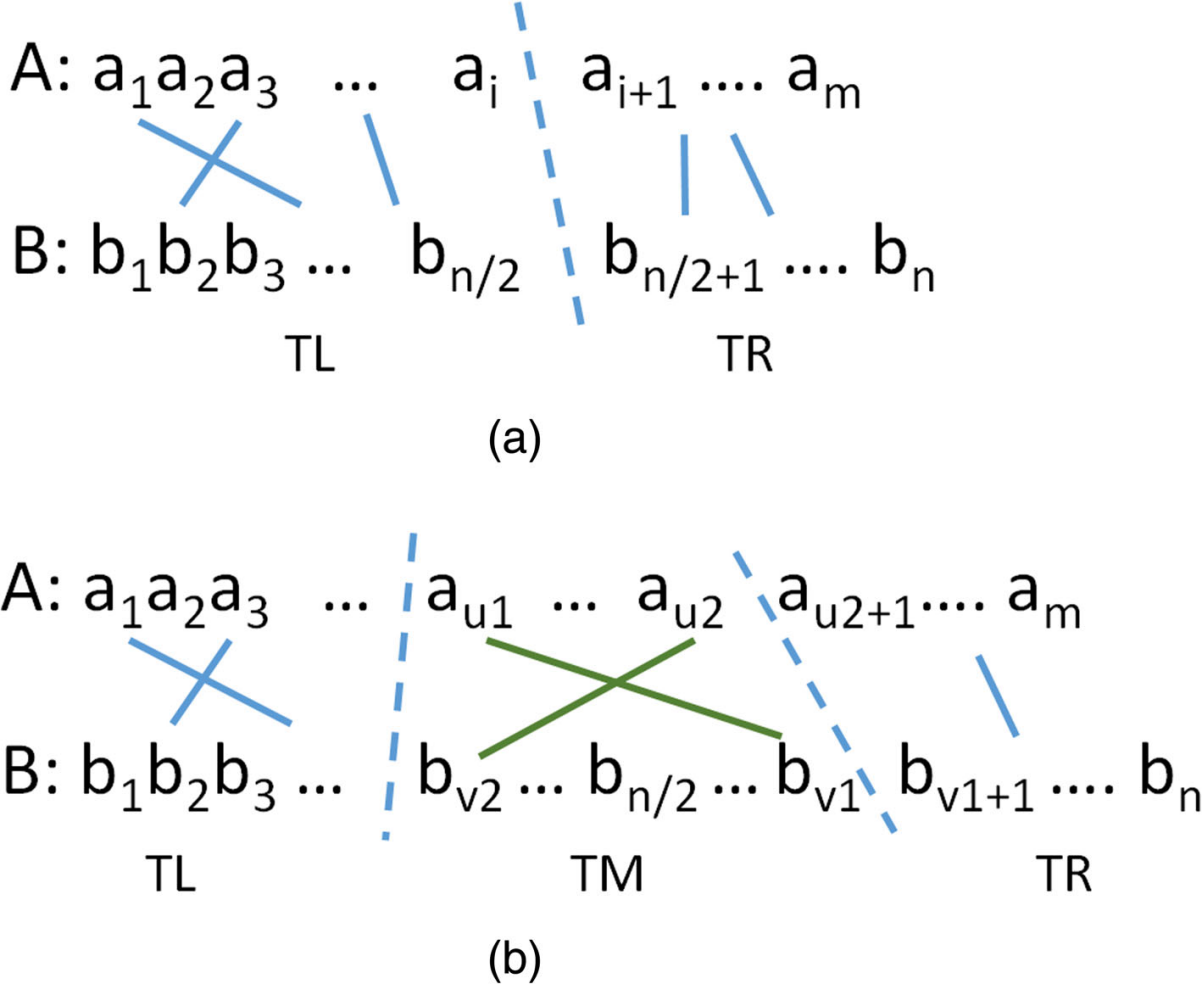
DL trace splitting opportunities. **a** No center crossing **b** With center crossing

We say that a trace has a *center crossing* iff it contains two lines (*u*_1_,*v*_1_) and (*u*_2_,*v*_2_), *u*_1_<*u*_2_ such that *v*_1_>*n*/2 and *v*_2_≤*n*/2 (Fig. 4).

Let *T* be an optimal trace that satisfies properties P2-P4. If *T* contains no center crossing, then its lines may be partitioned into sets *TL* and *TR* such that *TL* contains all lines (*u*,*v*)∈*T* with *v*≤*n*/2 and *TR* contains the remaining lines (Fig. 4a). Since there is no center crossing, all lines in *TR* have a *u* value greater than the *u* value of every line in *TL*. It follows from properties P2-P4 that there is an *i*, 1≤*i*≤*m* such that *T* is the union of an optimal trace for *A*[ 1:*i*] and *B*[ 1:*n*/2] and that for *A*[ *i*+1:*m*] and *B*[ *n*/2+1:*n*]. Let *H*[ *i*] be the cost the former optimal trace and *H*^′^[ *i*+1] that of the latter optimal trace. We see that when *T* has no center crossing, the cost of *T* is

3 $$ costNoCC(T) = \min_{1 \le i \le m}\{ H[\!i] + H'[\!i+1]\}  $$

When *T* contains a center crossing, its lines may be partitioned into 3 sets, *TL*, *TM*, and *TR*, as shown in Fig. 4b. Let (*u*_1_,*v*_1_) and (*u*_2_,*v*_2_) be the lines defining the center crossing. Note that *TL* contains all lines of *T* with *v*<*v*_2_, *TR* contains all lines with *v*>*v*_1_, and *TM*={(*u*_1_,*v*_1_),(*u*_2_,*v*_2_)}. Note also that all lines in *TL* have a *u*<*u*_1_ and all in *TR* have *u*>*u*_2_. From property P1, it follows that *TL* is an optimal trace for *A*[ 1:*u*_1_−1] and *B*[ 1:*v*_2_−1] and *TR* is an optimal trace for *A*[ *u*_2_+1:*m*] and *B*[ *v*_1_+1:*n*]. Further, since (*u*_1_,*v*_1_) and (*u*_2_,*v*_2_) are balanced lines, the cost of *TM* is (*u*_2_−*u*_1_−1)+1+(*v*_1_−*v*_2_−1). Also, *A*[ *u*_1_]≠*A*[ *u*_2_] as otherwise, replacing the center-crossing lines with (*u*_1_,*v*_2_) and (*u*_2_,*v*_1_) results in a lower cost trace. From property P4, we know that $u_{1} = lastA[\!u_{2}][\!b_{v_{1}}]\phantom {\dot {i}\!}$ and $v_{2} = lastB[\!v_{1}][\!a_{u_{2}}]\phantom {\dot {i}\!}$. Let *H*[ *i*][ *j*] be the cost of an optimal trace for *A*[ 1:*i*] and *B*[ 1:*j*] and let *H*^′^[ *i*][ *j*] be that for an optimal trace for *A*[ *i*:*m*] and *B*[ *j*:*n*]. So, when *T* has a center crossing, its cost is

4 $$  {\begin{aligned} costCC(T) \,=\,& \min\{H[\!u_{1}\,-\,1][\!v_{2}-1] + H'[\!u_{2}\!+1][\!v_{1}+1] \\ &+ (u_{2}-u_{1}-1) + 1 + (v_{1}-v_{2}-1)\} \end{aligned}}  $$

where, for the min{}, we try 1≤*u*_1_<*m* and for each such *u*_1_, we set *v*_1_ to be the smallest *i*>*n*/2 for which $b_{i} = a_{u_{1}}\phantom {\dot {i}\!}$. For each *u*_1_ we examine all characters other than $\phantom {\dot {i}\!}a_{u_{1}}$ in the alphabet. For each such character *c*, *v*_2_ is set to the largest *j*≤*n*/2 for which *b*_*j*_=*c* and *u*_2_ is the smallest *i*>*u*_1_ for which *a*_*i*_=*c*. So, the min is taken over (*s*−1)*m* terms.

Let *U*_*top*_ and *T*_*top*_ be the final *U* and *T* arrays computed by *LS*_*DL* with inputs *B*[ 1:*n*/2] and *A*[ 1:*m*] and *U*_*bot*_ and *T*_*bot*_ be these arrays when the inputs are the reverse of *B*[ *n*/2+1] and *A*[*m*:1]. From these arrays, we may readily determine the *H* and *H*^′^ values needed to evaluate Eqs. 3 and 4. Algorithm *LSDL*_*TRACE* (Algorithm 4) provides the pseudocode for our linear space computation of an optimal trace. It assumes that *LS*_*DL* has been modified to return both the arrays *U* and *T*.


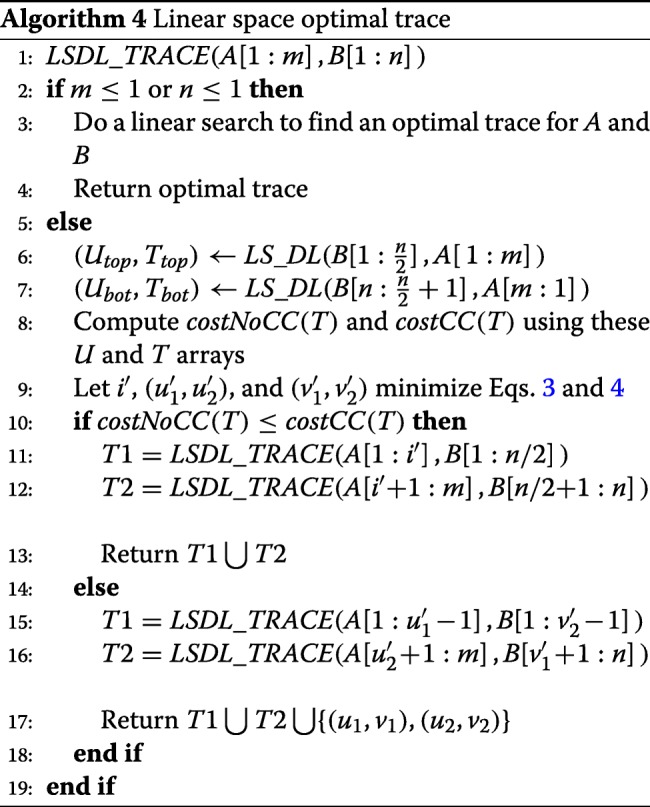


For the time complexity, we see that at the top level of the recursion, we invoke *LS*_*DL* twice with strings *A* and *B* of size *m* and *n*/2, respectively. This takes at most *amn* time for some constant *a*. The time required to compute Eqs. 3 and 4 is *O*(*sn*) and may be absorbed into *amn* by using a suitably large constant *a*. At the next level of recursion, *LS*_*DL* is invoked 4 times. The sum of the lengths of the *A* strings across these 4 invocations is at most 2*m* and the *B* string has length at most *n*/4. So, the time for these four invocations is at most *amn*/2. Generalizing to the remaining levels of recursion, we see that algorithm *LSDL*_*TRACE* takes *amn*(1+1/2+1/4+1/8+…)<2*amn*=*O*(*mn*) time. The space needed is the same as that for *LS*_*DL* (note that the parameters to this algorithm have been switched). From the time analysis, it follows that the number of cache misses is approximately twice that for *LS*_*DL* when invoked with strings of size *m* and *n*. Hence the approximate cache miss count for *LSDL*_*TRACE* is 2*mn*(1+3/*w*).

We note that some reduction in actual run time can be achieved by switching *A* and *B* when *A* is shorter than *B* thus ensuring that the shorter string is split at each level of recursion. This enables us to get the recursion terminates faster.

#### The strip trace algorithm *Strip*_*TRACE*

This algorithm differs from *LSDL*_*TRACE* in that it uses a modified version of *Strip*_*DL* rather than a modified version of *LS*_*DL*. The modified version of *Strip*_*DL* returns the arrays *strip* and *V* computed by *Strip*_*DL*. Correspondingly, *Strip*_*TRACE* uses *V*_*top*_ and *V*_*bot*_ in place of *T*_*top*_ and *T*_*bot*_. The asymptotic time complexity of *Strip*_*TRACE* is also *O*(*mn*) and it takes the same amount of space as does *Strip*_*DL* (note that the parameters to *Strip*_*DL* are switched relative to those for *Strip*_*TRACE*). The number of cache misses is approximately twice that for *Strip*_*DL*.

### Multi-core algorithms

In this section, we describe our parallelizations of algorithm *DL* and the four single-core algorithms of previous section. These parallelizations assume that the number of processors is small relative to string length. The naming convention we adopt for the parallel versions is adding *PP*_ as a prefix to the name of the single-core algorithm.

#### The algorithm *PP*_*DL*

Our parallel version of algorithm *DL*, *PP*_*DL*, computes the elements in the same order as does *DL*. However, it starts the computation of a row before the computation of its preceding row is complete. Each processor is assigned a unique row to compute and it computes this row from left to right. Let *p* be the number of processors. Processor *z* is initially assigned to do the outer loop computation for *i*=*z*, 1≤*i*≤*p*. Processor *z* begins after a suitable time lag relative to the start of processor *z*−1 so that the data it needs for its computation have already been computed by processor *z*−1. In our code, the time lag between the start of the computation of two consecutive rows is the time needed to compute *n*/*p* elements. Upon completion of its iteration *i* computation, the processor proceeds to iteration *i*+*p* of the outer loop. The time complexity of *PP*_*DL* is *O*(*mn*/*p*).

#### The algorithm *PP*_*LS*_*DL*

While the general parallelization strategy for *PP*_*LS*_*DL* is the same as that used in *PP*_*DL*, extra care is needed to ensure a computation identical to that of *LS*_*DL*. Divergence in results is possible when two or more processors are simultaneously computing different rows of *H* using the same memory. This happens for example when *A*=*aaabc*⋯ and *p*≥3. We start with processor *i* assigned to compute row *i* of *H*, 1≤*i*≤*p*. Suppose that *U*=*x* and *T*[ *a*]=*y* initially (note that *x* and *y* are addresses in memory). Because of the *swap*(*T*[ *A*[ *i*]],*U*) statement in *LS*_*DL*, processor 1 begins to compute row 1 of *H* using memory beginning at the address *y*. If processor 2 begins with a suitable time lag as in *PP*_*DL*, it will compute row 2 of *H* using memory beginning at the address *x*. With a further lag, processor 3 will begin to compute row 3 of *H* again using memory beginning at the address *y*. Now, both processors 1 and 3 are using the same memory to compute different rows of *H* and so we run the risk of overwriting *H* values that may be needed for subsequent computations. As another example, consider *A*=*ababa*⋯ and *p*≥4. Suppose that *U*=*x* and *T*[ *a*,*b*]=[ *y*,*z*] initially. Processor 1 begins to compute row 1 using the memory *y*, then, with a lag, processor 2 begins to compute row 2 using memory *z*, then processor 3 starts to compute row 3 using memory *x*. Next processor 4 begins to compute row 4 using memory *y*. At this time processor 1 is computing row 1 with *A*[ 1]=*a* and processor 4 is computing row 4 with *A*[ 4]=*b* and both processors are using the same row memory *y*.

Let *p*_1_ and *p*_2_ be two processors that are using the same memory to compute rows *r*_1_<*r*_2_ of *H* and that no processor is using this memory to compute a row between *r*_1_ and *r*_2_. From the swapping assignment scheme used in *LS*_*DL*, it follows that *p*_1_ is computing the row $r_{1} = lastA[\!r_{2}][\!a_{r_{2}}]-1\phantom {\dot {i}\!}$. The *H* values in this row are needed to compute rows *r*_1_+1 through *r*_2_ as $\phantom {\dot {i}\!}r_{1} = lastA[\!i][\!a_{r_{2}}] r_{1} < i \le r_{2}$. These values are not needed for rows *i*>*r*_2_ as for these rows $\phantom {\dot {i}\!}lastA[\!i][\!a_{r_{2}}] = r_{2} > r_{1} + 1 = lastA[\!r_{2}][\!a_{r_{2}}]$. Let *j*_1_ be such that $\phantom {\dot {i}\!}b_{j} = a_{r_{2}} = a_{r_{1} + 1}$. Then, for *j*>*j*_1_, $\phantom {\dot {i}\!}lastB[\!j][\!a_{r_{2}}] \ge j_{1}$. Hence, for *j*>*j*_1_ columns 1 through *j*_1_−2 of row *r*_1_ are not needed to compute an *H* in rows between *r*_1_ and *r*_2_.

Our parallel code uses a synchronization scheme that is based on the observations of the preceding paragraph to delay the overwriting of values that are needed for later computations and ensure a correct computation of the DL distance. Our synchronization scheme employs another array *W*[ 1:*n*] that is initialized to 1. Suppose that a processor is computing row *i* of *H* and that *A*[ *i*]=*a*. When this processor first encounters an *a* in *B*, say at position *j*_1_, it increments *W*[0:*j*_1_−2]. When the next *a* isencountered, say at *j*_2_, it increments *W*[ *j*_1_−1:*j*_2_−2] by 1. When the processor finishes its computation of row *i*, the remaining positions of *W* are incremented by 1. The processor assigned to compute row *q* of *H* may compute *U*[ *j*] iff *W*[ *j*]=*q*. From our earlier observations, it follows that when *W*[ *j*]=*q*, the old values in memory positions *U*[ 1:*j*] may be overwritten as these are not needed for future computations.

This *p*-processor algorithm *PP*_*LS*_*DL*’s time complexity depends on the data sets as the synchronization delay is data dependent. We, however, expect a run-time performance of approximately *O*(*mn*/*p*) when the characters in *B* are roughly uniformly distributed.

#### The algorithm *PP*_*Strip*_*DL*

In the parallel version *PP*_*Strip*_*DL* of *Strip*_*DL*, processor *i* is initially assigned to compute strip *i*, 1≤*i*≤*p*. Upon completion of its currently assigned strip *j*, the processor proceeds to compute strip *j*+*p*. An array *signal*[] is used for synchronization purposes. When computing a row *r* in its assigned strip *s*, a processor needs to wait until *signal*[ *r*]=*s*. *signal*[ *r*] is set to *s* by the processor working on strip *s*−1 when the values to the left of strip *s* needed in the computation of row *r* of strip *s* have been computed and there is no risk that the computations for row *r* of strip *s* will overwrite *V* values needed by other processors. *signal* works very much like *W* in *PP*_*LS*_*DL*.

Note that when we are working on *p* strips, we need *p* copies of the arrays *U* and *T* used by *Strip*_*DL*.

The time complexity of *PP*_*Strip*_*DL* depends on the synchronization delay and is expected to approximate *O*(*mn*/*p*).

#### The algorithm *PP*_*DL*_*TRACE*

This algorithm first uses *PP*_*DL* to compute *H*[][]. Then, a single processor performs a traceback to construct the optimal trace. For reasonable values of *p*, the run time is dominated by *PP*_*DL* and so, the complexity of *PP*_*DL*_*TRACE* is also *O*(*mn*/*p*).

#### The algorithms *PP*_*LSDL*_*TRACE* and *PP*_*Strip*_*TRACE*

In *LSDL*_*TRACE* (*Strip*_*TRACE*), we repeatedly partition the problem into two and apply either *LS*_*DL* (*Strip*_*DL*) to each partition. The parallel version *PP*_*LSDL*_*TRACE* (*PP*_*Strip*_*TRACE*) employs the following parallelization strategy:

- Each subproblem is solved using *PP*_*LS*_*DL* (*PP*_*Strip*_*DL*) when the number of independent subproblems is small; all *p* processors are assigned to the parallel solution of a single subproblem. I.e., the subproblems are solved in sequence.
- *p* subproblems are solved in parallel using *LS*_*DL* (*Strip*_*DL*) to solve each subproblem serially when the number of independent subproblems is large,

The time complexity of *PP*_*LSDL*_*TRACE* and *PP*_*Strip*_*TRACE* is *O*(*mn*/*p*).

## Results

### Experimental platform and test data

The single-core algorithms were implemented using C and the multi-core ones using C and OpenMP. Our codes may be downloaded from [23]. The following computational platforms were used:

1. Xeon4: Intel Xeon CPU E5-2603 v2 Quad-Core processor 1.8GHz with 10MB cache and 32GB memory.
2. Xeon6: Intel I7-x980 Six-Core processor 3.33GHz with 12MB LLC cache and 16GB memory.
3. Xeon24: Intel Xeon CPU E5-2695 v2 2xTwelve-Core processors 2.40GHz with 30MB cache and 512GB memory.

We compiled all codes using the gcc compiler with the O2 option. Cache miss and energy consumption data were obtained for our Xeon4 platform using the “perf” [24] software and the RAPL interface. This is the only platform for which we obtained cache miss and energy consumption data.

For test data, we downloaded the real DNA/RNA/protein sequences from the NCBI (National Center for Biotechnology Information) server [25] and PDB (Protein Data Bank) server [15]. In addition to that, we also generated random DNA/RNA and protein sequences.

### Xeon E5-2603 (Xeon4) using random data

#### DL distance algorithms

**Fig. 5:**
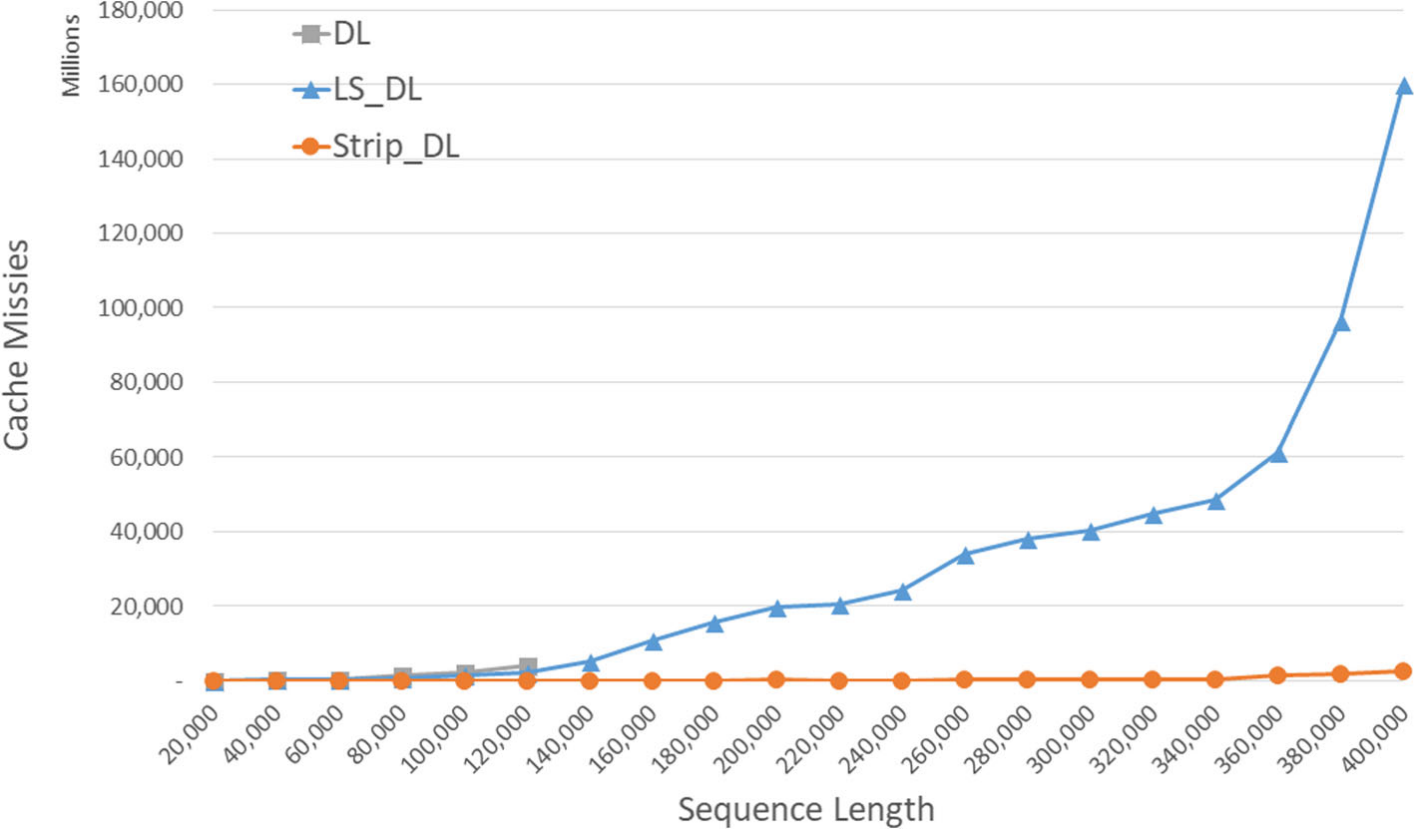
Cache misses for DL distance algorithms on Xeon4

The observed cache misses for our DL distance algorithms on our Xeon4 platform for randomly generated sequences of size between 40000 and 400000 are given in Fig. 5 and Table 1. “**” in the table indicates there was insufficient memory for the algorithm to run. The column of Table 1 labeled *LvsD* (*SvsD*) presents the percentage changes in cache misses reduced by *LS*_*DL* (*Strip*_*DL*) relative to *DL* while that labeled *SvsL* gives this percentage changes reduced by *Strip*_*DL* relative to *LS*_*DL*.

**Table 1:** Cache misses for DL distance algorithms, in millions, on Xeon4

A	B	DL	LS_DL	Strip_DL	L vs D	S vs D	S vs L
40000	40000	201	265	6	-31.8%	97.5%	97.9%
80000	80000	1267	715	16	43.6%	98.8%	97.8%
120000	120000	4006	2180	42	45.6%	99.0%	98.1%
160000	160000	**	10,652	63			99.4%
200000	200000	**	19,751	147			99.3%
240000	240000	**	24,257	133			99.5%
280000	280000	**	38,119	188			99.5%
320000	320000	**	44,815	242			99.5%
360000	360000	**	61,296	1352			97.8%
400000	400000	**	160,118	2407			98.5%

^**^ ⇒ insufficient memory

Notice that *DL* runs out of memory when |*A*|=|*B*|≥160000. *Strip*_*DL* has fewer cache misses than *LS*_*DL* and *LS*_*DL* has fewer cache misses than *DL*. *Strip*_*DL* reduces cache misses by up to 99.0*%* relative to *DL* and by up to 99.5*%* relative to *LS*_*DL*.

**Fig. 6:**
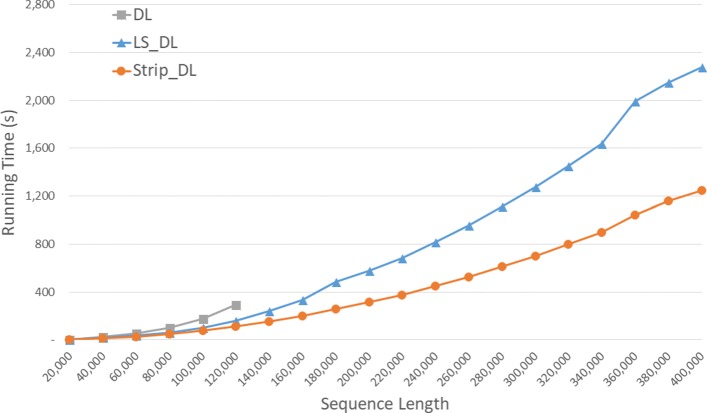
Run time of DL distance algorithms, in seconds, on Xeon4

Run times are given in seconds in Fig. 6 and using the format *hh*:*mm*:*ss* in Table 2 for our random data set. *Strip*_*DL* is the fastest followed by *LS*_*DL* and *DL*. *Strip*_*DL* reduces run time by up to 61.3*%* relative to *DL* and by up to 47.6*%* relative to *LS*_*DL*.

**Table 2:** Run time of DL distance algorithms on Xeon4

A	B	DL	LS_DL	Strip_DL	L vs D	S vs D	S vs L
40000	40000	0:00:27	0:00:17	0:00:13	34.3%	53.0%	28.4%
80000	80000	0:01:40	0:01:02	0:00:50	37.8%	50.1%	19.7%
120000	120000	0:04:50	0:02:40	0:01:52	44.8%	61.3%	29.9%
160000	160000	**	0:05:37	0:03:19			40.8%
200000	200000	**	0:09:38	0:05:14			45.7%
240000	240000	**	0:13:37	0:07:28			45.1%
280000	280000	**	0:18:34	0:10:10			45.2%
320000	320000	**	0:24:13	0:13:17			45.1%
360000	360000	**	0:33:10	0:17:22			47.6%
400000	400000	**	0:37:55	0:20:46			45.3%

**Fig. 7:**
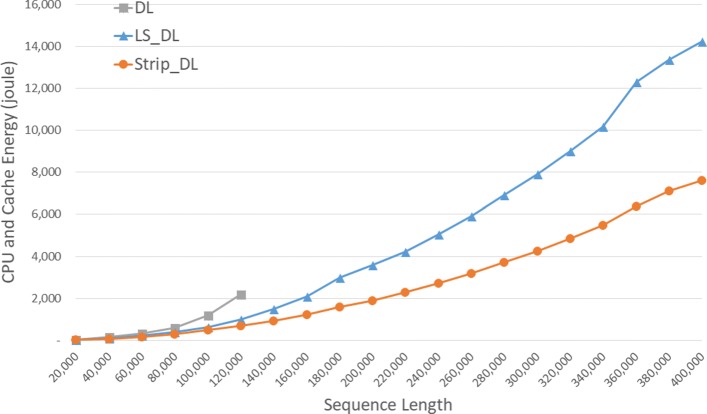
CPU and cache energy consumption of DL distance algorithms, in joules, on Xeon4

Energy consumption by the CPU and cache are gievn, in joules, in Fig. 7 and Table 3. *Strip*_*DL* required up to 68.5*%* less CPU and cache energy than *DL* and up to 48.2*%* less than *LS*_*DL*.

**Table 3:** CPU and cache energy consumption of DL distance algorithms on Xeon4

A	B	DL	LS_DL	Strip_DL	L vs D	S vs D	S vs L
40000	40000	158.77	107.1	76.73	32.5%	51.7%	28.4%
80000	80000	598.12	383.88	305.12	35.8%	49.0%	20.5%
120000	120000	2180.59	996.9	686.54	54.3%	68.5%	31.1%
160000	160000	**	2088.01	1212.27			41.9%
200000	200000	**	3576.52	1905.54			46.7%
240000	240000	**	5058.27	2714.47			46.3%
280000	280000	**	6905.74	3711.18			46.3%
320000	320000	**	9000.26	4852.4			46.1%
360000	360000	**	12286.83	6365.86			48.2%
400000	400000	**	14218.28	7615.16			46.4%

#### DL trace algorithms

**Fig. 8:**
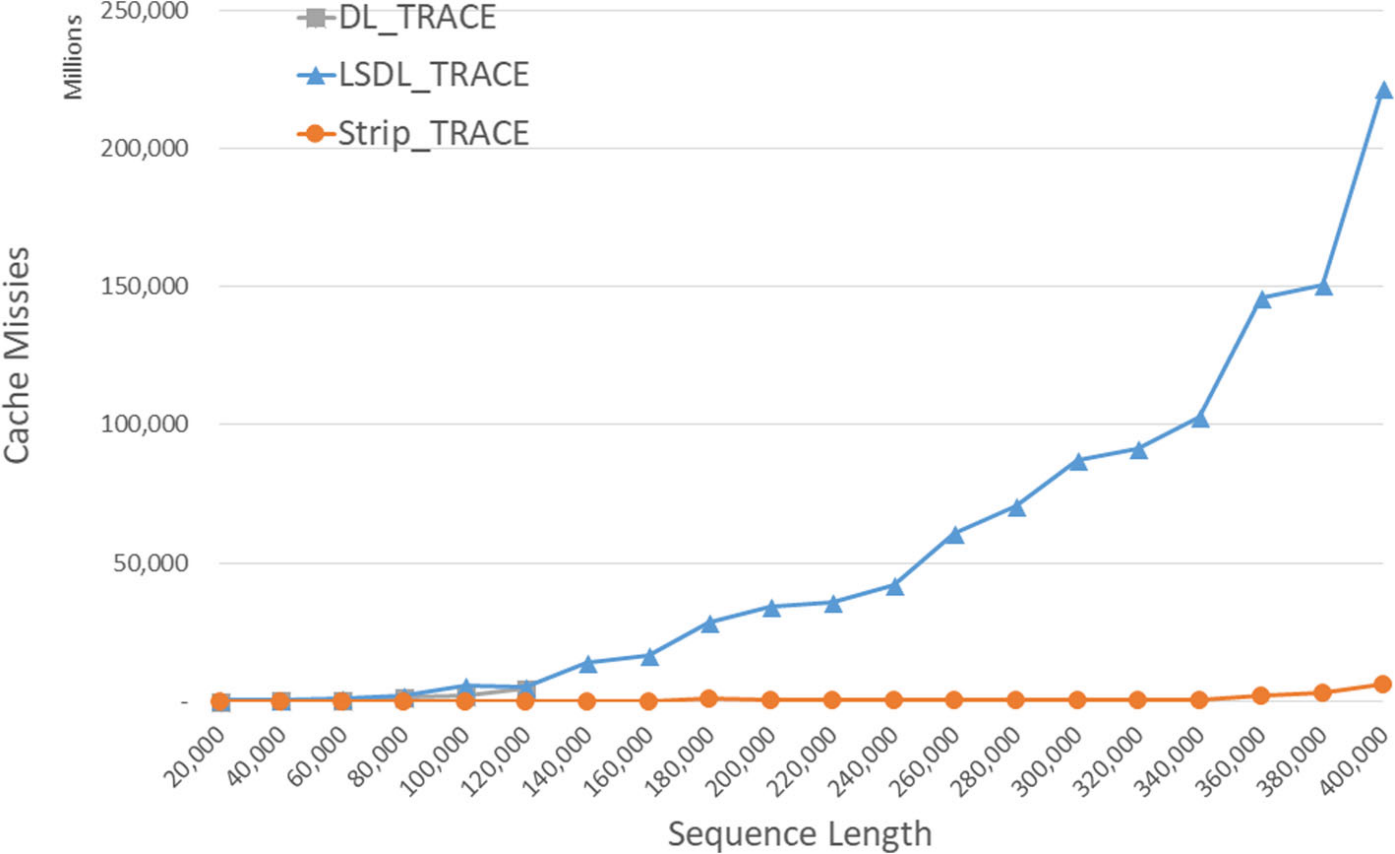
Cache misses for DL trace algorithms on Xeon4

The observed cache misses for our single-core DL trace algorithms on our Xeon4 platform are given in Fig. 8 and Table 4. Since *DL*_*TRACE* is simply *DL* with a linear time traceback added, that cache miss count for *DL*_*TRACE* is only slightly more than that for *DL*. *LSDL*_*TRACE* has a higher count than does *DL*_*TRACE* for the instances that *DL* has sufficient memory to solve though the gap narrows with increasing instance size. *Strip*_*TRACE* consistently has fewer cache misses than both *DL*_*TRACE* and *LSDL*_*TRACE*. *Strip*_*TRACE* reduces cache misses by up to 98.6*%* relative to *DL*_*TRACE* and by up to 99.5*%* relative to *LSDL*_*TRACE*.

**Table 4:** Cache misses for DL trace algorithms, in millions, on Xeon4

A	B	DL_TRACE	LSDL_TRACE	Strip_TRACE	L vs D	S vs D	S vs L
40000	40000	220	423	24	-92.0%	89.3%	94.4%
80000	80000	1537	1970	29	-28.1%	98.1%	98.5%
120000	120000	4852	5100	66	-5.1%	98.6%	98.7%
160000	160000	**	16,350	115			99.3%
200000	200000	**	33,998	513			98.5%
240000	240000	**	42,252	268			99.4%
280000	280000	**	70,370	358			99.5%
320000	320000	**	91,501	453			99.5%
360000	360000	**	146,103	2120			98.5%
400000	400000	**	221,690	6032			97.3%

**Fig. 9:**
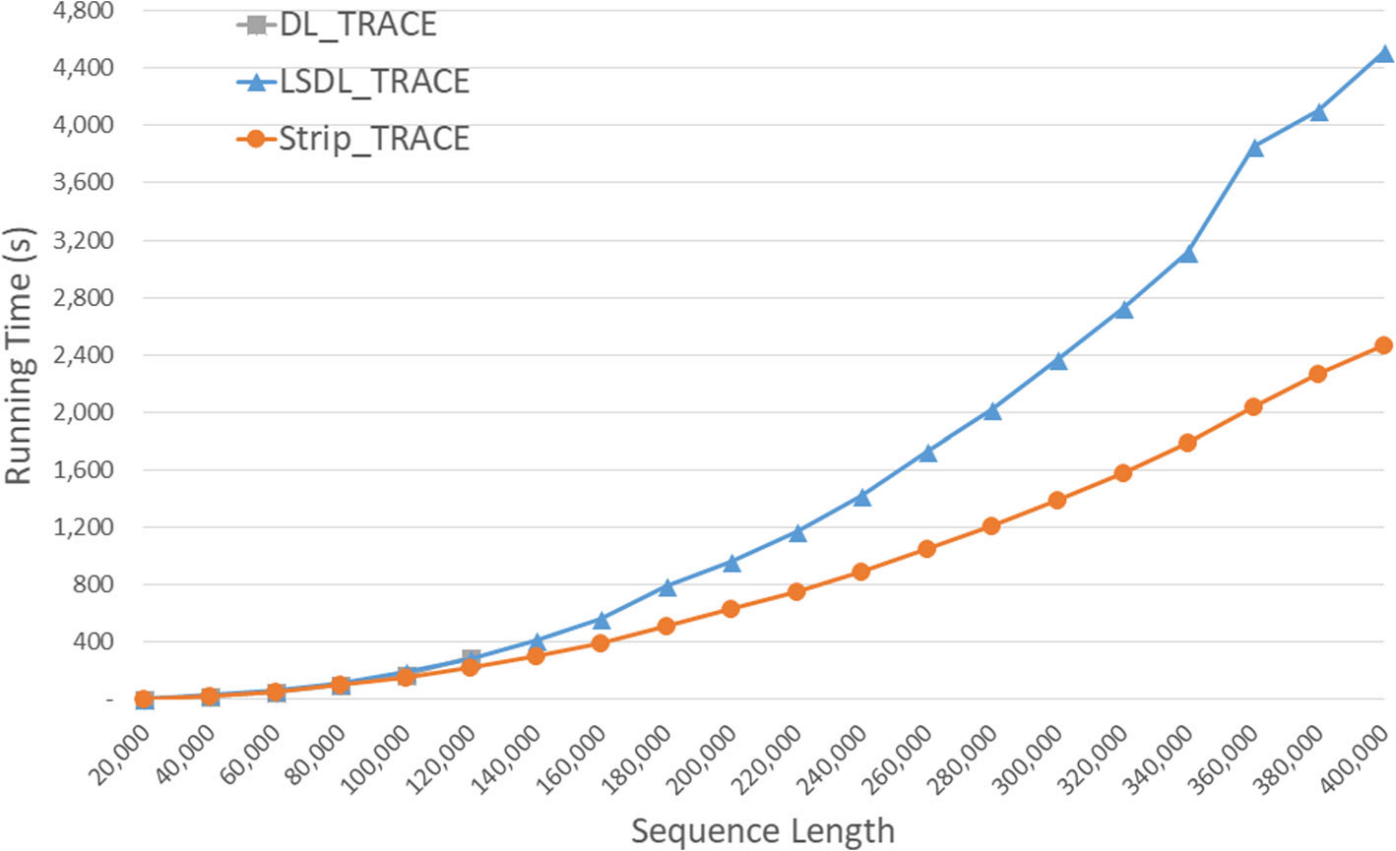
Run time of DL trace algorithms, in seconds, on Xeon4

Run times of the DL trace algorithms on our Xeon4 platform are given in seconds in Fig. 9 and Table 5. *Strip*_*TRACE* is competitive with *DL*_*TRACE* on our instances of size 40,000 and 80,000 and 23.5% faster on the instance of size 120,000. *Strip*_*TRACE* was consistently faster than *LSDL*_*TRACE* achieving a speedup of up to 47.1*%*.

**Table 5:** Run time of DL trace algorithms on Xeon4

A	B	DL_TRACE	LSDL_TRACE	Strip_TRACE	L vs D	S vs D	S vs L
40000	40000	0:00:27	0:00:30	0:00:26	-11.3%	3.5%	13.3%
80000	80000	0:01:40	0:01:54	0:01:40	-14.5%	-0.4%	12.4%
120000	120000	0:04:53	0:04:42	0:03:44	3.6%	23.5%	20.6%
160000	160000	**	0:09:21	0:06:37			29.2%
200000	200000	**	0:15:58	0:10:30			34.2%
240000	240000	**	0:23:42	0:14:52			37.3%
280000	280000	**	0:33:41	0:20:13			40.0%
320000	320000	**	0:45:26	0:26:24			41.9%
360000	360000	**	1:04:18	0:34:01			47.1%
400000	400000	**	1:15:14	0:41:11			45.3%

**Fig. 10:**
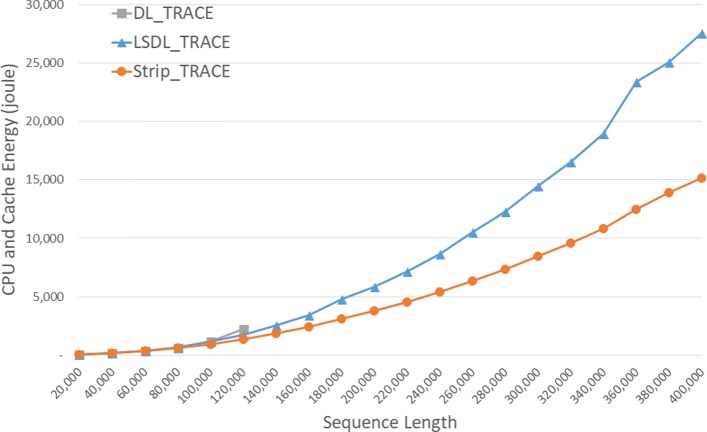
CPU and cache energy consumption of DL trace algorithms, in joules, on Xeon4

The energy consumed by the CPU and cache is given in Fig. 10 and Table 6. *Strip*_*TRACE* required up to 46.8*%* less CPU and cache energy than *LSDL*_*TRACE*.

**Table 6:** CPU and cache energy consumption of DL trace algorithms on Xeon4

A	B	DL_TRACE	LSDL_TRACE	Strip_TRACE	L vs D	S vs D	S vs L
40000	40000	158.56	181.40	156.95	-14.4%	1.0%	13.5%
80000	80000	597.27	703.09	610.70	-17.7%	-2.2%	13.1%
120000	120000	2,256.99	1,736.86	1,365.32	23.0%	39.5%	21.4%
160000	160000	**	3,443.83	2,407.86			30.1%
200000	200000	**	5,843.56	3,818.68			34.7%
240000	240000	**	8,665.30	5,403.60			37.6%
280000	280000	**	12,275.03	7,372.25			39.9%
320000	320000	**	16,536.93	9,609.56			41.9%
360000	360000	**	23,396.41	12,439.71			46.8%
400000	400000	**	27,551.90	15,167.76			44.9%

#### Parallel DL distance algorithms

**Fig. 11:**
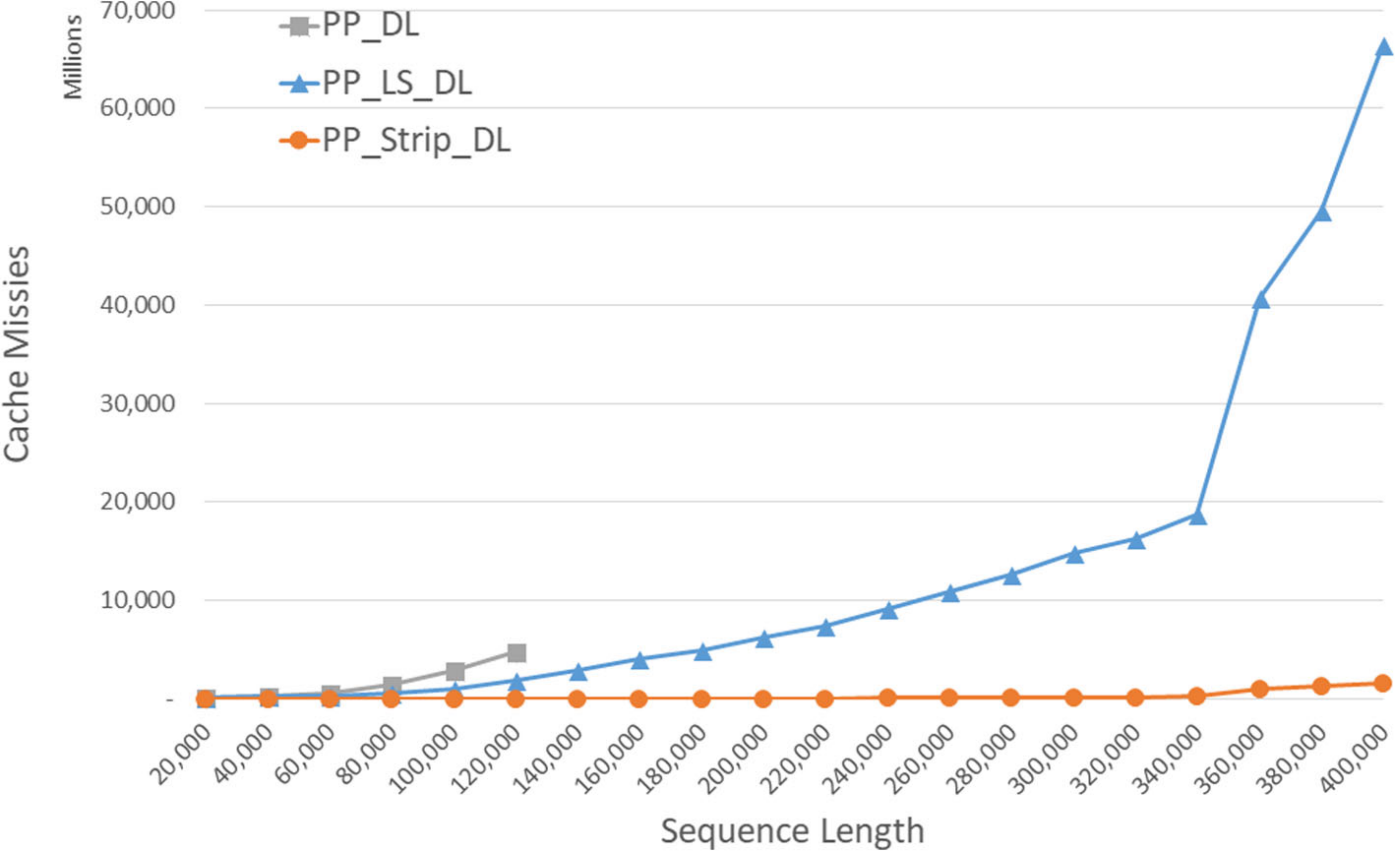
Cache misses of parallel DL distance algorithms on Xeon4

The observed cache misses for our parallel DL algorithms are given in Fig. 11 and Table 7. *PP*_*Strip*_*DL* has the fewest cache misses followed by *PP*_*LS*_*DL* and *PP*_*DL* (in this order). The reduction is cache misses achieved by *PP*_*Strip*_*DL* is up to 99.6*%* relative to *PP*_*DL* and up to 99.4*%* relative to *PP*_*LS*_*DL*.

**Table 7:** Cache misses of parallel DL distance algorithms, in millions, on Xeon4

A	B	PP_DL	PP_LS_DL	PP_Strip_DL	L vs D	S vs D	S vs L
40000	40000	259	235	3	9.2%	99.0%	98.9%
80000	80000	1417	500	6	64.7%	99.6%	98.8%
120000	120000	4746	1857	24	60.9%	99.5%	98.7%
160000	160000	**	4028	26			99.4%
200000	200000	**	6243	43			99.3%
240000	240000	**	9101	66			99.3%
280000	280000	**	12,636	112			99.1%
320000	320000	**	16,267	202			98.8%
360000	360000	**	40,741	1020			97.5%
400000	400000	**	66,469	1644			97.5%

**Fig. 12:**
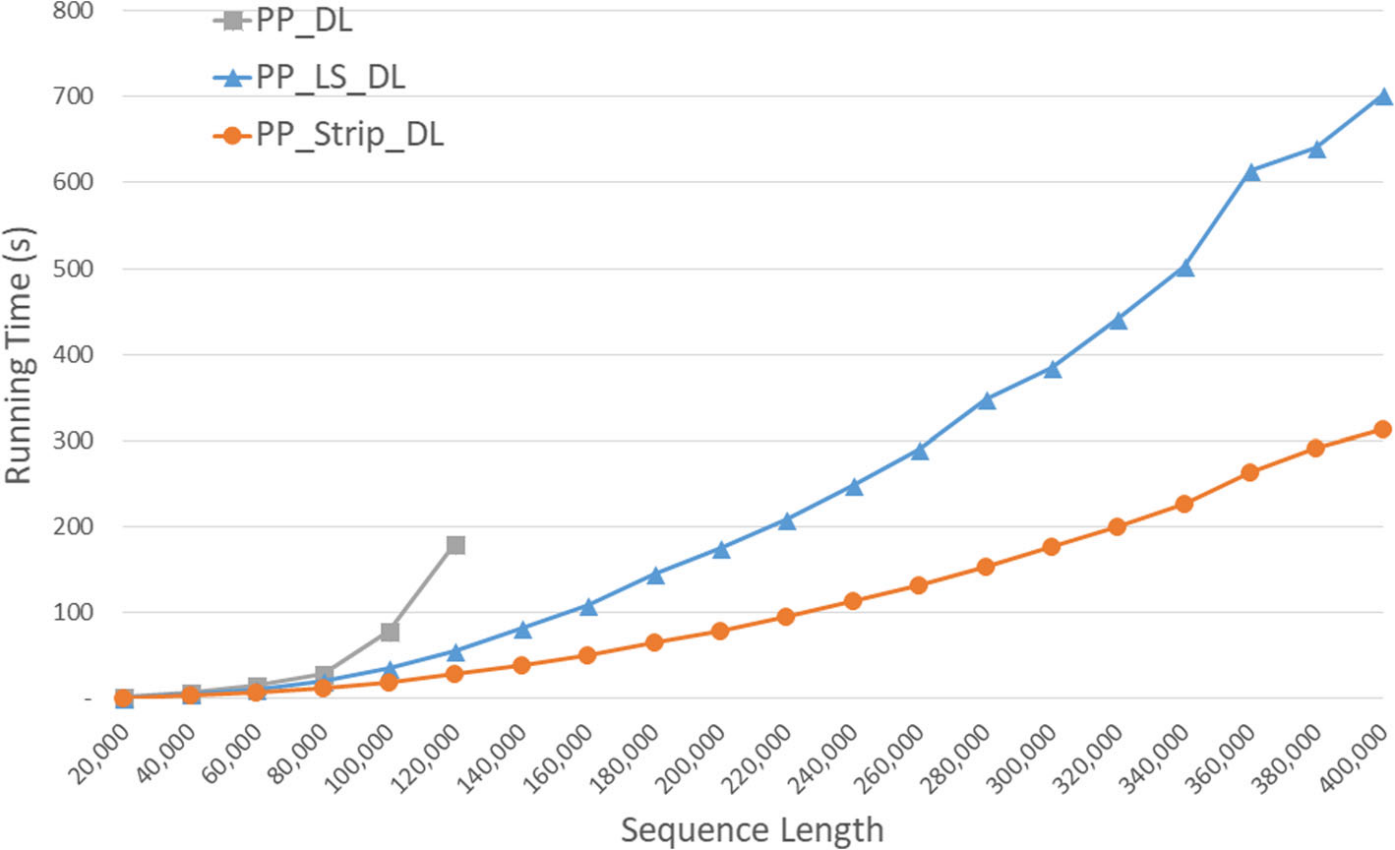
Run time of parallel DL distance algorithms, in seconds, on Xeon4

Run times for our parallel DL algorithms are given in Fig. 12 and Table 8. *PP*_*Strip* is up to 84.2*%* faster than *PP*_*DL* and up to 57.0*%* faster than *PP*_*LS*_*DL*.

**Table 8:** Run time of parallel DL distance algorithms on Xeon4

A	B	PP_DL	PP_LS_DL	PP_Strip_DL	L vs D	S vs D	S vs L
40000	40000	0:00:08	0:00:05	0:00:03	33.4%	59.0%	38.4%
80000	80000	0:00:29	0:00:20	0:00:13	30.7%	56.7%	37.5%
120000	120000	0:03:00	0:00:56	0:00:28	68.9%	84.2%	49.2%
160000	160000	**	0:01:49	0:00:50			54.1%
200000	200000	**	0:02:55	0:01:19			55.2%
240000	240000	**	0:04:09	0:01:53			54.5%
280000	280000	**	0:05:48	0:02:34			55.9%
320000	320000	**	0:07:21	0:03:20			54.5%
360000	360000	**	0:10:13	0:04:24			57.0%
400000	400000	**	0:11:41	0:05:13			55.3%

Speedup numbers are given in Table 9. The column labeled *DL*/*PP*, for example, is the time for *DL* divided by that for *PP*_*DL*. *PP*_*Strip*_*DL* has a speedup between 3.95 and 3.99, which is quite close to the number of cores (4) on our Xeon4 platform. The speedup for *PP*_*DL* is up to 3.45 and that for *PP*_*LS*_*DL* is up to 3.40.

**Table 9:** Speedup of parallel DL distance algorithms on Xeon4

A	B	DL/PP	LS_DL/PP	Strip_DL/PP
40000	40000	3.45	3.40	3.96
80000	80000	3.44	3.09	3.97
120000	120000	1.62	2.87	3.96
160000	160000	**	3.08	3.98
200000	200000	**	3.30	3.99
240000	240000	**	3.29	3.96
280000	280000	**	3.20	3.97
320000	320000	**	3.30	3.98
360000	360000	**	3.24	3.95
400000	400000	**	3.24	3.98

**Fig. 13:**
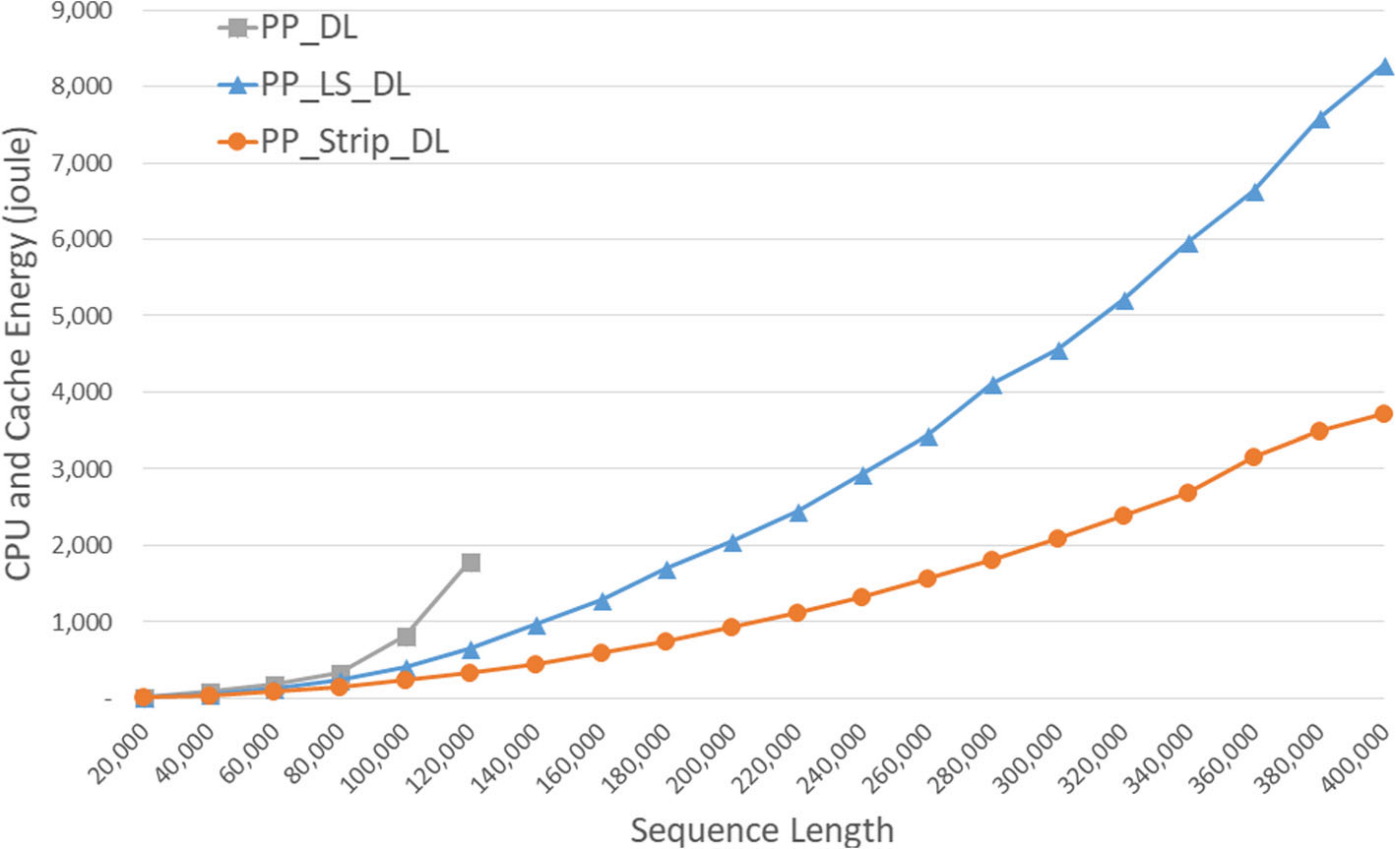
CPU and cache energy consumption of parallel DL distance algorithms, in joules, on Xeon4

Energy data are given in Fig. 13 and Table 10. *PP*_*Strip*_*DL* used up to 81.4*%* less CPU and cache energy than did *PP*_*DL* and up to 55.7*%* less than *PP*_*LS*_*DL*.

**Table 10:** CPU and cache energy consumption of parallel DL distance algorithms on Xeon4

A	B	PP_DL	PP_LS_DL	PP_Strip_DL	L vs D	S vs D	S vs L
40000	40000	89.12	60.64	37.31	32.0%	58.1%	38.5%
80000	80000	336.87	238.15	147.82	29.3%	56.1%	37.9%
120000	120000	1800.48	657.89	334.86	63.5%	81.4%	49.1%
160000	160000	**	1285.34	591.9			53.9%
200000	200000	**	2063.55	926.64			55.1%
240000	240000	**	2928.87	1332.29			54.5%
280000	280000	**	4106.15	1818.66			55.7%
320000	320000	**	5223.54	2385.45			54.3%
360000	360000	**	6640.93	3164.4			52.4%
400000	400000	**	8287.46	3727.31			55.0%

Although the multi-core algorithms use more CPU power than used by their single-core counterparts, the power increase is less than the decrease in run time. Hence, energy consumption is reduced.

#### Parallel DL trace algorithms

**Fig. 14:**
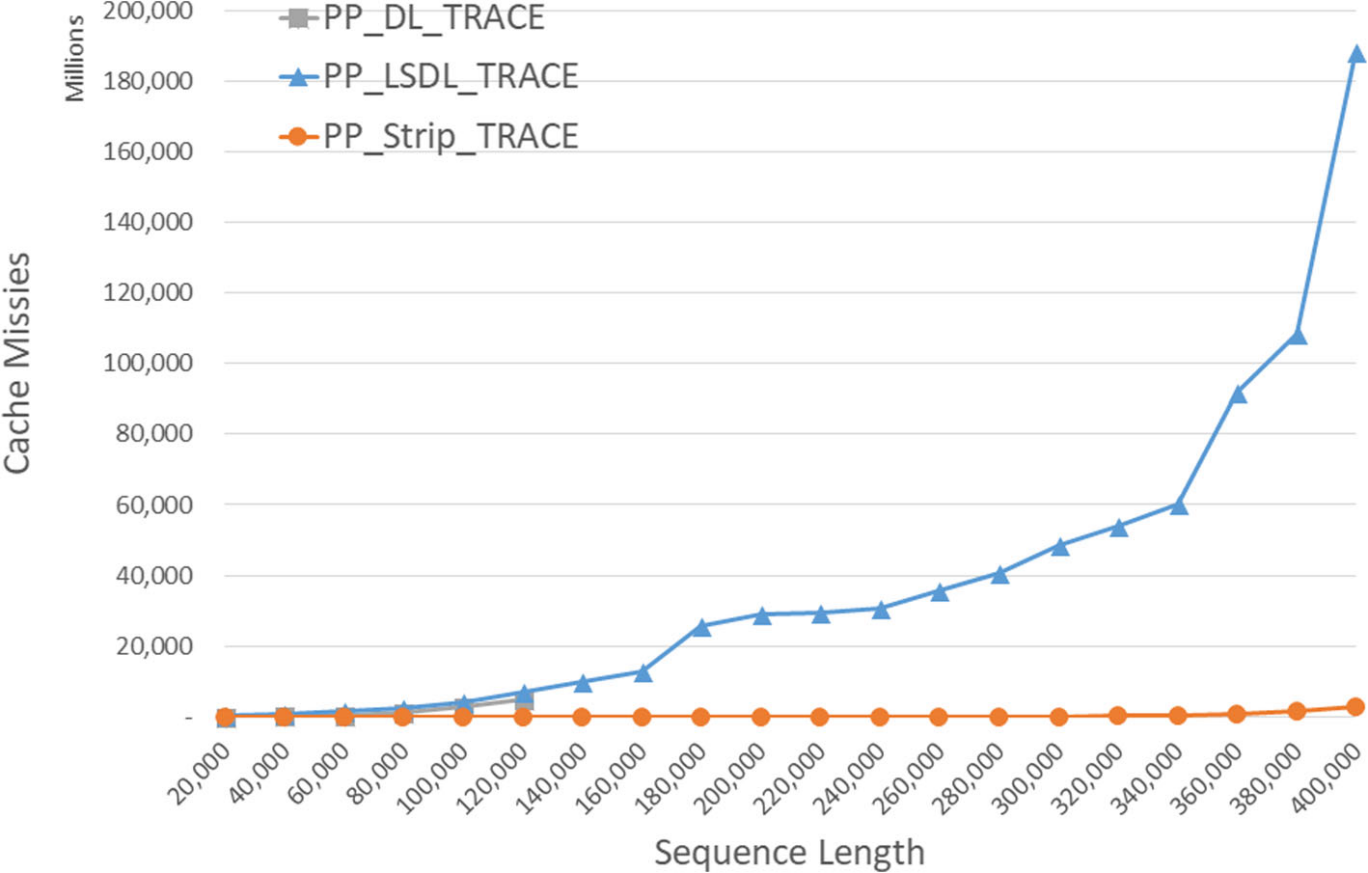
Cache misses for parallel DL trace algorithms on Xeon4

The number of cache misses incurred by our multi-core DL trace algorithms is given in Fig. 14 and Table 11. *PP*_*Strip*_*TRACE* has the fewest number of cache misses. *PP*_*Strip*_*TRACE* reduces cache misses by up to 99.3*%* and 99.6*%* relative to *PP*_*DL*_*TRACE* and *PP*_*LSDL*_*TRACE*, respectively.

**Table 11:** Cache misses for parallel DL trace algorithms, in millions, on Xeon4

A	B	PP_DL_TRACE	PP_LSDL_TRACE	PP_Strip_TRACE	L vs D	S vs D	S vs L
40000	40000	247	636	16	-157.0%	93.6%	97.5%
80000	80000	1312	2431	15	-85.3%	98.9%	99.4%
120000	120000	4940	6814	34	-37.9%	99.3%	99.5%
160000	160000	**	12,774	53			99.6%
200000	200000	**	28,908	85			99.7%
240000	240000	**	30,529	110			99.6%
280000	280000	**	40,803	154			99.6%
320000	320000	**	53,892	179			99.7%
360000	360000	**	91,621	796			99.1%
400000	400000	**	188,325	2727			98.6%

**Fig. 15:**
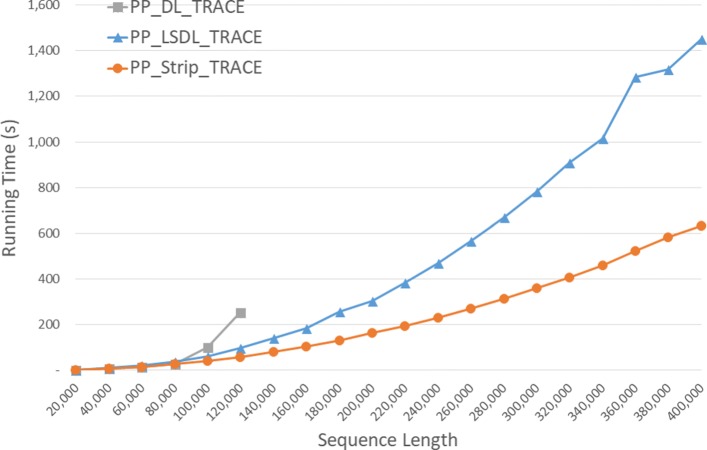
Run time of parallel DL trace algorithms, in seconds, on Xeon4

Run times are given in Fig. 15 and Table 12. *PP*_*Strip*_*Trace* is faster than *PP*_*LSDL*_*TRACE* by up to 59.2*%*. As in Table 13, the speedup achieved by *PP*_*Strip*_*TRACE* relative to its single-core version ranges from 3.44 to 3.90.

**Table 12:** Run time of parallel DL trace algorithms on Xeon4

A	B	PP_DL_TRACE	PP_LSDL_TRACE	PP_Strip_TRACE	L vs D	S vs D	S vs L
40000	40000	0:00:08	0:00:10	0:00:07	-36.1%	0.9%	27.2%
80000	80000	0:00:29	0:00:38	0:00:27	-32.7%	7.0%	29.9%
120000	120000	0:04:15	0:01:39	0:00:59	61.2%	76.8%	40.3%
160000	160000	**	0:03:05	0:01:43			44.2%
200000	200000	**	0:05:03	0:02:43			46.3%
240000	240000	**	0:07:50	0:03:50			51.0%
280000	280000	**	0:11:11	0:05:13			53.4%
320000	320000	**	0:15:07	0:06:46			55.2%
360000	360000	**	0:21:25	0:08:44			59.2%
400000	400000	**	0:24:10	0:10:34			56.3%

**Table 13:** Speedup of parallel DL trace algorithms on Xeon4

A	B	DL/PP	LSDL_TRACE/PP	Strip_TRACE/PP
40000	40000	3.53	2.88	3.44
80000	80000	3.44	2.97	3.72
120000	120000	1.15	2.86	3.79
160000	160000	**	3.03	3.85
200000	200000	**	3.16	3.87
240000	240000	**	3.02	3.87
280000	280000	**	3.01	3.88
320000	320000	**	3.01	3.90
360000	360000	**	3.00	3.89
400000	400000	**	3.11	3.90

**Fig. 16:**
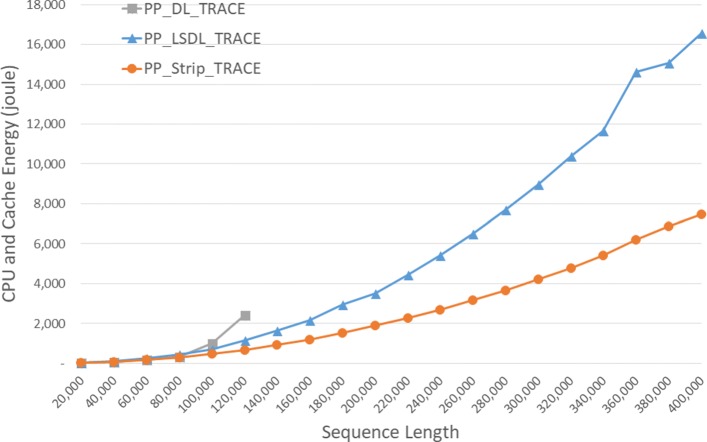
CPU and cache energy consumption of parallel DL trace algorithms, in joules, on Xeon4

Energy consumption data are given in Fig. 16 and Table 14. *PP*_*Strip*_*TRACE* required up to 57.6*%* less CPU and cache energy than *PP*_*LSDL*_*TRACE*.

**Table 14:** CPU and cache energy consumption of parallel DL trace algorithms on Xeon4

A	B	PP_DL_TRACE	PP_LSDL_TRACE	PP_Strip_TRACE	L vs D	S vs D	S vs L
40000	40000	87.39	118.74	84.85	-35.9%	2.9%	28.5%
80000	80000	334.01	449.89	310.34	-34.7%	7.1%	31.0%
120000	120000	2,433.28	1,149.61	684.28	52.8%	71.9%	40.5%
160000	160000	**	2,149.58	1,202.52			44.1%
200000	200000	**	3,524.59	1,898.35			46.1%
240000	240000	**	5,410.42	2,684.72			50.4%
280000	280000	**	7,707.41	3,657.54			52.5%
320000	320000	**	10,384.75	4,789.03			53.9%
360000	360000	**	14,612.39	6,200.10			57.6%
400000	400000	**	16,559.76	7,472.52			54.9%

#### Xeon E5-2603 (Xeon4) using real data

Tables 15 and 16, respectively, give the run times for our single-core and multi-core DL and DL trace algorithms using real DNA sequences on our Xeon4 platform. The observed times are quite comparable to those for similarly sized random strings. Further, the speed up achieved by our parallel algorithms relative to the single-core algorithms is also comparable to that for random strings. So, for our remaining test platforms, we present only the results for our randomly generated data sets.

**Table 15:** Run time of DL distance algorithms for real DNA sequences on Xeon4

A	B	DL	LS_DL	Strip_DL	PP_DL	PP_LS_DL	PP_Strip_DL
NZ_LRIA01000064	CYPR01000097	0:00:23	0:00:19	0:00:12	0:00:08	0:00:06	0:00:03
LNFE01000131	AGUF01000028	0:01:27	0:01:16	0:00:47	0:00:29	0:00:24	0:00:12
NZ_CYTG01000018	LVKN01000071	0:03:21	0:02:53	0:01:46	0:02:42	0:00:54	0:00:28
BX000446	BX511181	**	0:05:01	0:03:09	**	0:01:34	0:00:49
NZ_AMFW01000007	LYHN01000016	**	0:07:49	0:04:54	**	0:02:26	0:01:17
JLXA01000008	AUHZ01000004	**	0:11:31	0:07:04	**	0:03:38	0:01:51
NZ_FNNC01000004	NZ_APZF01000097	**	0:15:03	0:09:37	**	0:04:43	0:02:31
LHOK01000008	AGYI01000018	**	0:19:44	0:12:36	**	0:06:11	0:03:17
BAMV01000017	MIMZ01000025	**	0:24:56	0:15:56	**	0:07:49	0:04:09
LSMI01000030	CZBU01000005	**	0:30:13	0:19:39	**	0:09:34	0:05:08

**Table 16:** Run time of DL trace algorithms for real DNA sequences on Xeon4

A	B	DL_Trace	LSDL_Trace	Strip_Trace	PP_DL_Trace	PP_LSDL_Trace	PP_Strip_Trace
NZ_LRIA01000064	CYPR01000097	0:00:23	0:00:33	0:00:24	0:00:08	0:00:12	0:00:08
LNFE01000131	AGUF01000028	0:01:27	0:02:12	0:01:35	0:00:29	0:00:45	0:00:28
NZ_CYTG01000018	LVKN01000071	0:03:23	0:05:02	0:03:33	0:03:48	0:01:44	0:01:00
BX000446	BX511181	**	0:08:45	0:06:18	**	0:02:59	0:01:45
NZ_AMFW01000007	LYHN01000016	**	0:13:43	0:09:51	**	0:04:39	0:02:43
JLXA01000008	AUHZ01000004	**	0:20:10	0:14:10	**	0:06:50	0:03:54
NZ_FNNC01000004	NZ_APZF01000097	**	0:26:25	0:19:18	**	0:08:49	0:05:17
LHOK01000008	AGYI01000018	**	0:34:41	0:25:15	**	0:11:29	0:06:55
BAMV01000017	MIMZ01000025	**	0:43:49	0:31:58	**	0:14:38	0:08:44
LSMI01000030	CZBU01000005	**	0:52:54	0:39:23	**	0:18:14	0:10:46

### I7-x980 (Xeon6) using random data

#### DL distance algorithms

**Fig. 17:**
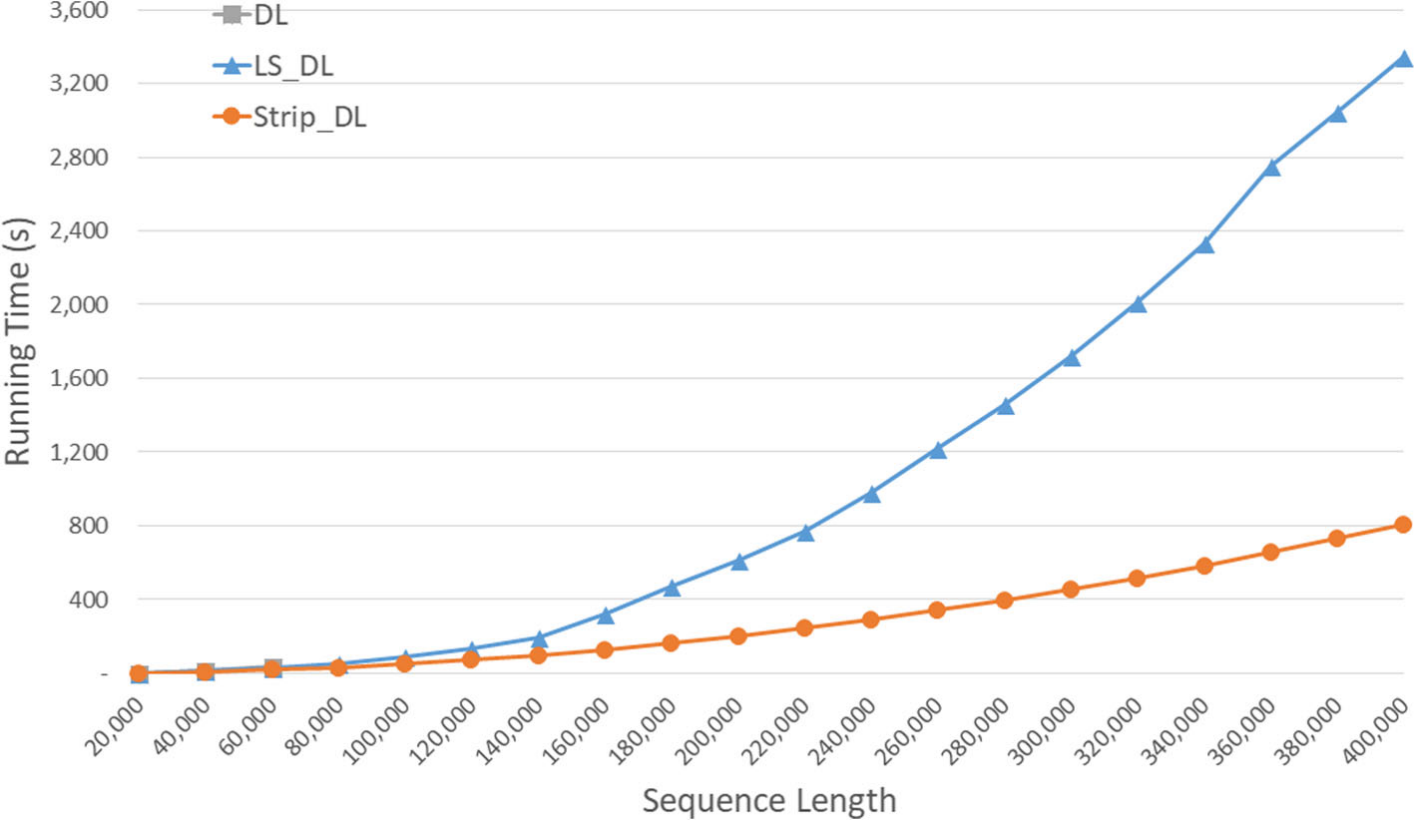
Run time of DL distance algorithms, in seconds, on Xeon6

Single core run times are given in Fig. 17 and Table 17 for our Xeon6 platform. As can be seen, *Strip*_*DL* is the fastest followed by *LS*_*DL* and *DL*. *Strip*_*DL* reduces run time by up to 52.3*%* relative to *DL* and by up to 76.1*%* relative to *LS*_*DL*. The classical *DL* algorithm ran out memory when |*A*|=|*B*|=8000.

**Table 17:** Run time of DL distance algorithms on Xeon6

A	B	DL	LS_DL	Strip_DL	L vs D	S vs D	S vs L
40000	40000	0:00:17	0:00:14	0:00:08	16.2%	52.3%	43.1%
80000	80000	**	0:00:55	0:00:32			41.3%
120000	120000	**	0:02:12	0:01:13			44.8%
160000	160000	**	0:05:19	0:02:09			59.5%
200000	200000	**	0:10:16	0:03:23			67.1%
240000	240000	**	0:16:17	0:04:50			70.3%
280000	280000	**	0:24:19	0:06:36			72.9%
320000	320000	**	0:33:32	0:08:36			74.4%
360000	360000	**	0:45:50	0:10:58			76.1%
400000	400000	**	0:55:44	0:13:27			75.9%

#### DL trace algorithms

**Fig. 18:**
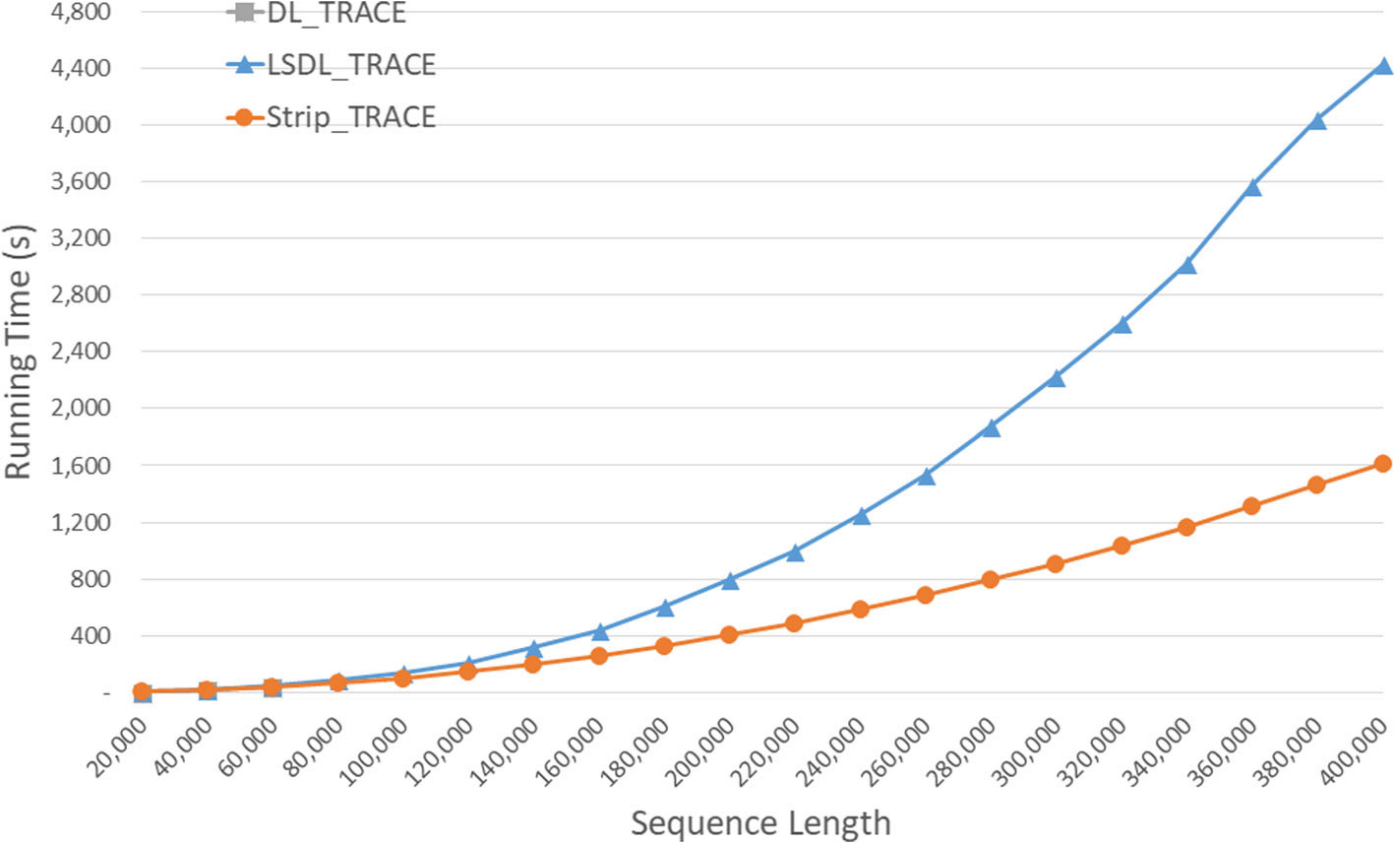
Run time of DL trace algorithms, in seconds, on Xeon6

The run times for the DL trace algorithms are given in Fig. 18 and Table 18. *Strip*_*TRACE* reduces run time by up to 63.5*%* relative to *LSDL*_*TRACE*.

**Table 18:** Run time of DL trace algorithms on Xeon6

A	B	DL_TRACE	LSDL_TRACE	Strip_TRACE	L vs D	S vs D	S vs L
40000	40000	0:00:17	0:00:22	0:00:17	-26.3%	4.8%	24.6%
80000	80000	**	0:01:24	0:01:05			22.1%
120000	120000	**	0:03:33	0:02:26			31.2%
160000	160000	**	0:07:20	0:04:20			41.0%
200000	200000	**	0:13:19	0:06:46			49.1%
240000	240000	**	0:20:51	0:09:43			53.4%
280000	280000	**	0:31:19	0:13:14			57.7%
320000	320000	**	0:43:24	0:17:16			60.2%
360000	360000	**	0:59:27	0:21:55			63.1%
400000	400000	**	1:13:51	0:26:57			63.5%

#### Parallel DL distance algorithms

**Fig. 19:**
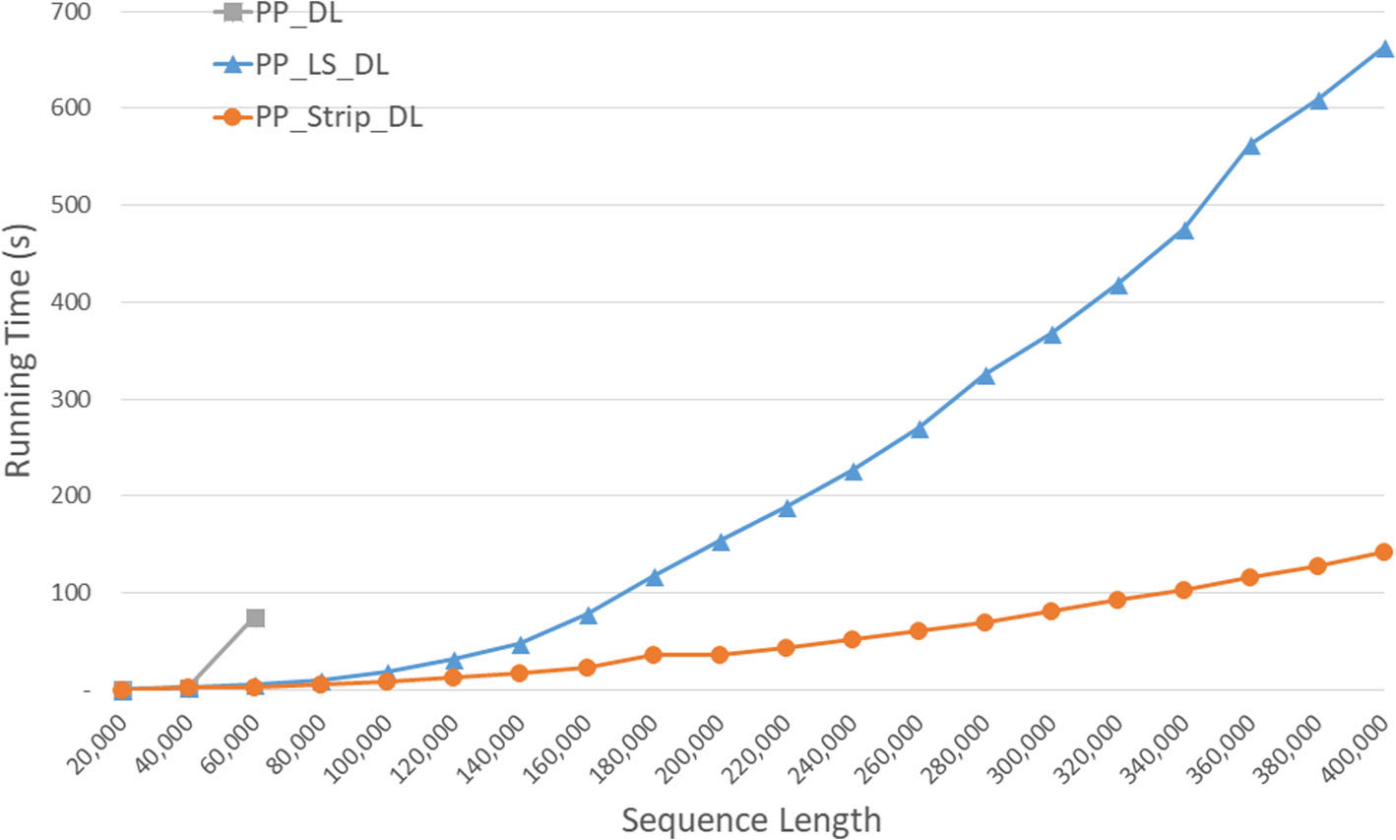
Run time of parallel DL distance algorithms, in seconds, on Xeon6

Run times for the parallel DL distance algorithms are given in Fig. 19 and Table 19. As was the case on our *Xeon*4 platform, *PP*_*Strip*_*DL* is faster than *PP*_*DL* and *PP*_*LS*_*DL*. It reduces the run time by up to 25.6*%* and 79.4*%*, respectively. The speedup of our parallel algorithm *PP*_*Strip*_*DL* relative to its single-core version (Table 20) is up to 5.71. This is quite close to the number of cores (6). The maximum speedup achieved by *PP*_*DL* and *PP*_*LS*_*DL* was 4.89 and 5.27, respectively.

**Table 19:** Run time of parallel DL distance algorithms on Xeon6

A	B	PP_DL	PP_LS_DL	PP_Strip_DL	L vs D	S vs D	S vs L
40000	40000	0:00:03	0:00:03	0:00:03	22.2%	25.6%	4.4%
80000	80000	**	0:00:11	0:00:06			46.7%
120000	120000	**	0:00:32	0:00:13			59.8%
160000	160000	**	0:01:18	0:00:23			71.1%
200000	200000	**	0:02:34	0:00:36			76.8%
240000	240000	**	0:03:47	0:00:52			77.2%
280000	280000	**	0:05:25	0:01:09			78.6%
320000	320000	**	0:06:59	0:01:32			77.9%
360000	360000	**	0:09:23	0:01:56			79.4%
400000	400000	**	0:11:03	0:02:23			78.5%

**Table 20:** Speedup of parallel DL distance algorithms on Xeon6

A	B	DL/PP	LS_DL/PP	Strip_DL/PP
40000	40000	4.89	5.27	3.13
80000	80000	**	5.15	5.68
120000	120000	**	4.08	5.59
160000	160000	**	4.07	5.71
200000	200000	**	3.99	5.67
240000	240000	**	4.30	5.60
280000	280000	**	4.49	5.70
320000	320000	**	4.80	5.58
360000	360000	**	4.88	5.67
400000	400000	**	5.04	5.65

#### Parallel DL trace algorithms

**Fig. 20:**
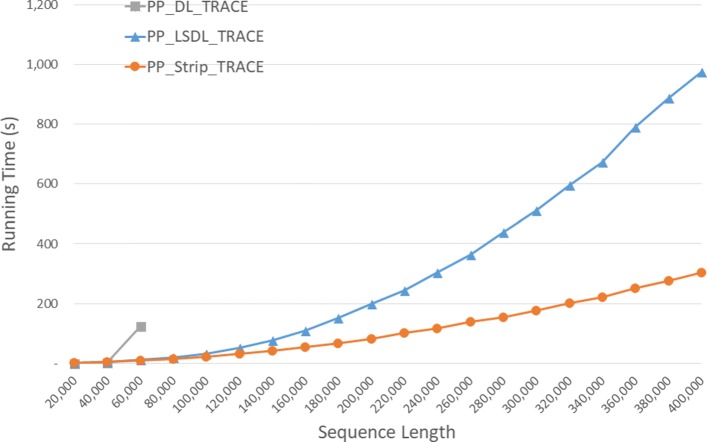
Run time of parallel DL trace algorithms, in seconds, on Xeon6

Xeon6 run times for the parallel DL trace algorithms are given in Fig. 20 and Table 21. *PP*_*Strip*_*TRACE* is faster than *PP*_*LSDL*_*TRACE* and reduces the run time by up to 68.9*%*. As shown in Table 22, *PP*_*Strip*_*TRACE* obtains a speedup of up to 5.33 while the maximum speedup by *PP*_*DL*_*TRACE* and *PP*_*LSDL*_*TRACE* was 5.23 and 4.55, respectively.

**Table 21:** Run time of parallel DL trace algorithms on Xeon6

A	B	PP_DL_TRACE	PP_LSDL_TRACE	PP_Strip_TRACE	L vs D	S vs D	S vs L
40000	40000	0:00:03	0:00:05	0:00:05	-56.2%	-51.2%	3.2%
80000	80000	**	0:00:19	0:00:16			15.7%
120000	120000	**	0:00:51	0:00:31			38.1%
160000	160000	**	0:01:49	0:00:56			48.9%
200000	200000	**	0:03:19	0:01:21			59.3%
240000	240000	**	0:05:04	0:01:56			61.9%
280000	280000	**	0:07:19	0:02:33			65.1%
320000	320000	**	0:09:55	0:03:21			66.3%
360000	360000	**	0:13:11	0:04:12			68.2%
400000	400000	**	0:16:14	0:05:03			68.9%

**Table 22:** Speedup of parallel DL trace algorithms on Xeon6

A	B	DL_TRACE/PP	LSDL_TRACE/PP	Strip_TRACE/PP
40000	40000	5.23	4.23	3.30
80000	80000	**	4.49	4.15
120000	120000	**	4.19	4.65
160000	160000	**	4.05	4.68
200000	200000	**	4.02	5.02
240000	240000	**	4.12	5.04
280000	280000	**	4.28	5.18
320000	320000	**	4.38	5.17
360000	360000	**	4.51	5.22
400000	400000	**	4.55	5.33

### Xeon E5-2695 (Xeon24) using random data

#### DL distance algorithms

**Fig. 21:**
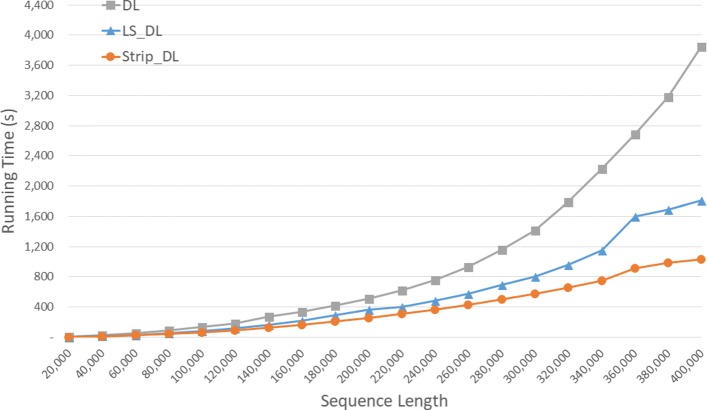
Run time of DL distance algorithms, in seconds, on Xeon24

The run times for our single-core DL distance algorithms on the Xeon24 are given in Fig. 21 and Table 23. As on our other test platforms, *Strip*_*DL* is the fastest followed by *LS*_*DL* and *DL*. *Strip*_*DL* reduces run time by up to 73.1*%* relative to *DL* and by up to 42.9*%* relative to *LS*_*DL*.

**Table 23:** Run time of DL distance algorithms on Xeon24

A	B	DL	LS_DL	Strip_DL	L vs D	S vs D	S vs L
40000	40000	0:00:24	0:00:14	0:00:10	41.5%	57.1%	26.7%
80000	80000	0:01:26	0:00:53	0:00:41	38.3%	53.0%	23.8%
120000	120000	0:03:06	0:02:01	0:01:31	35.3%	50.9%	24.2%
160000	160000	0:05:33	0:03:34	0:02:42	35.6%	51.3%	24.3%
200000	200000	0:08:32	0:06:08	0:04:15	28.2%	50.2%	30.6%
240000	240000	0:12:40	0:08:05	0:06:05	36.2%	52.0%	24.8%
280000	280000	0:19:24	0:11:32	0:08:20	40.6%	57.0%	27.7%
320000	320000	0:29:51	0:15:55	0:10:58	46.7%	63.3%	31.1%
360000	360000	0:44:44	0:26:41	0:15:11	40.4%	66.0%	43.1%
400000	400000	1:04:03	0:30:11	0:17:15	52.9%	73.1%	42.9%

#### DL trace algorithms

**Fig. 22:**
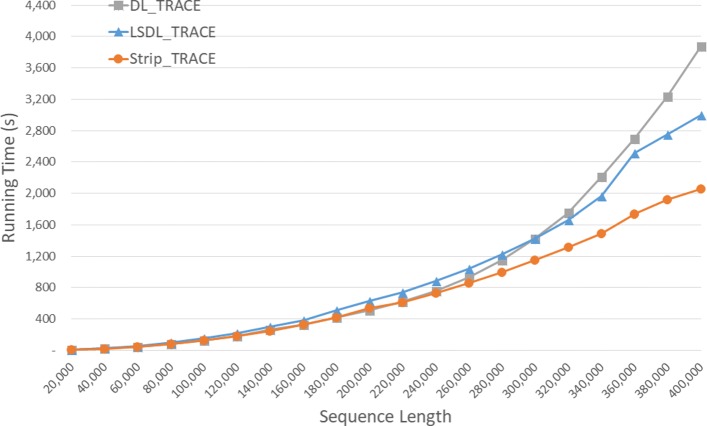
Run time of DL trace algorithms, in seconds, on Xeon24

The run times for our single-core DL trace algorithms on the Xeon24 are given in Fig. 22 and Table 24. *Strip*_*TRACE* reduces run time by up to 46.9*%* and 31.5*%* relative to *DL*_*TRACE* and *LSDL*_*TRACE*, respectively.

**Table 24:** Run time of DL trace algorithms on Xeon24

A	B	DL_TRACE	LSDL_TRACE	Strip_TRACE	L vs D	S vs D	S vs L
40000	40000	0:00:23	0:00:25	0:00:21	-6.1%	9.7%	14.9%
80000	80000	0:01:25	0:01:36	0:01:23	-12.8%	2.7%	13.8%
120000	120000	0:03:04	0:03:38	0:03:04	-18.2%	-0.1%	15.3%
160000	160000	0:05:29	0:06:26	0:05:27	-17.1%	0.7%	15.2%
200000	200000	0:08:30	0:10:26	0:08:54	-22.9%	-4.8%	14.7%
240000	240000	0:12:40	0:14:44	0:12:14	-16.3%	3.5%	17.0%
280000	280000	0:19:07	0:20:22	0:16:39	-6.5%	12.9%	18.2%
320000	320000	0:29:14	0:27:39	0:21:51	5.4%	25.3%	21.0%
360000	360000	0:44:52	0:41:56	0:28:55	6.6%	35.6%	31.0%
400000	400000	1:04:33	0:50:01	0:34:16	22.5%	46.9%	31.5%

**Table 25:** Run time of parallel DL distance algorithms on Xeon24

A	B	PP_DL	PP_LS_DL	PP_Strip_DL	L vs D	S vs D	S vs L
40000	40000	0:00:02	0:00:01	0:00:01	67.7%	74.7%	21.5%
80000	80000	0:00:08	0:00:04	0:00:02	53.6%	75.1%	46.4%
120000	120000	0:00:16	0:00:08	0:00:04	51.8%	72.7%	43.5%
160000	160000	0:00:28	0:00:18	0:00:08	34.8%	70.4%	54.6%
200000	200000	0:00:44	0:00:28	0:00:11	37.1%	74.6%	59.6%
240000	240000	0:01:04	0:00:37	0:00:16	42.0%	74.3%	55.8%
280000	280000	0:01:33	0:00:59	0:00:23	36.3%	75.8%	61.9%
320000	320000	0:02:11	0:01:33	0:00:30	29.1%	77.4%	68.1%
360000	360000	0:03:07	0:02:01	0:00:40	35.1%	78.5%	66.9%
400000	400000	0:03:34	0:02:44	0:00:45	23.2%	79.1%	72.8%

**Table 26:** Speedup of parallel DL distance algorithms on Xeon24

A	B	DL/PP	LS_DL/PP	Strip_DL/PP
40000	40000	9.86	17.88	16.69
80000	80000	10.53	13.99	19.90
120000	120000	11.39	15.29	20.51
160000	160000	12.02	11.86	19.77
200000	200000	11.51	13.14	22.57
240000	240000	11.91	13.10	22.29
280000	280000	12.51	11.68	22.19
320000	320000	13.70	10.30	22.27
360000	360000	14.39	13.21	22.73
400000	400000	18.00	11.05	23.22

#### Parallel DL trace algorithms

Parallel DL trace run times are given in Fig. 24 and Table 27. *PP*_*Strip*_*TRACE* is faster than *PP*_*DL*_*TRACE* and *PP*_*LSDL*_*TRACE* on large data. It reduces the run time by up to 51.1*%* and 50.1*%*, respectively. *PP*_*Strip*_*TRACE* achieves a speedup of up to 17.42 (Table 28); *PP*_*DL*_*TRACE* and *PP*_*LSDL*_*TRACE* have maximum speedups of 16.98 and 12.60.

**Table 27:** Run time of parallel DL trace algorithms on Xeon24

A	B	PP_DL_TRACE	PP_LSDL_TRACE	PP_Strip_TRACE	L vs D	S vs D	S vs L
40000	40000	0:00:01	0:00:03	0:00:02	-153.0%	-79.8%	28.9%
80000	80000	0:00:07	0:00:11	0:00:07	-58.3%	-7.1%	32.3%
120000	120000	0:00:17	0:00:21	0:00:15	-25.4%	8.3%	26.9%
160000	160000	0:00:30	0:00:35	0:00:24	-18.4%	18.1%	30.8%
200000	200000	0:00:47	0:00:53	0:00:41	-11.0%	14.3%	22.8%
240000	240000	0:01:08	0:01:13	0:00:53	-8.0%	21.7%	27.5%
280000	280000	0:01:39	0:01:52	0:01:10	-13.4%	28.9%	37.3%
320000	320000	0:02:27	0:02:30	0:01:26	-1.8%	41.7%	42.7%
360000	360000	0:03:23	0:03:20	0:01:40	1.5%	50.9%	50.1%
400000	400000	0:04:22	0:04:14	0:02:08	3.3%	51.1%	49.5%

**Table 28:** Speedup of parallel DL trace algorithms on Xeon24

A	B	DL_TRACE/PP	LSDL_TRACE/PP	Strip_TRACE/PP
40000	40000	16.98	7.12	8.53
80000	80000	12.59	8.97	11.43
120000	120000	11.01	10.38	12.02
160000	160000	11.14	11.02	13.51
200000	200000	10.76	11.91	13.15
240000	240000	11.20	12.06	13.81
280000	280000	11.65	10.94	14.26
320000	320000	11.92	11.08	15.28
360000	360000	13.29	12.60	17.42
400000	400000	14.77	11.83	16.04

#### Parallel DL distance algorithms

**Fig. 23:**
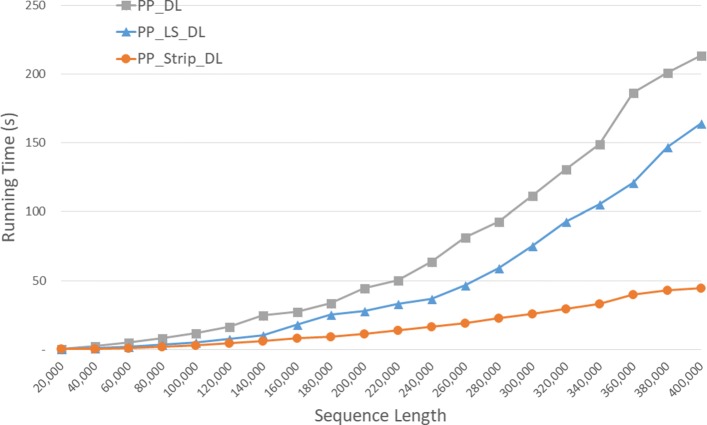
Run time of parallel DL distance algorithms, in seconds, on Xeon24

Parallel DL distance run times are given in Fig. 23 and Table 25. *PP*_*Strip*_*DL* is faster than *PP*_*DL* and *PP*_*LS*_*DL* and reduces the run time by up to 79.1*%* and 72.8*%*, respectively. As can be seen from Table 26, *PP*_*Strip*_*DL* scales quite well and results in a speedup of up to 23.22. The maximum speedups provided by *PP*_*DL* and *PP*_*LS*_*DL* are 18.00 and 17.88, respectively.

**Fig. 24:**
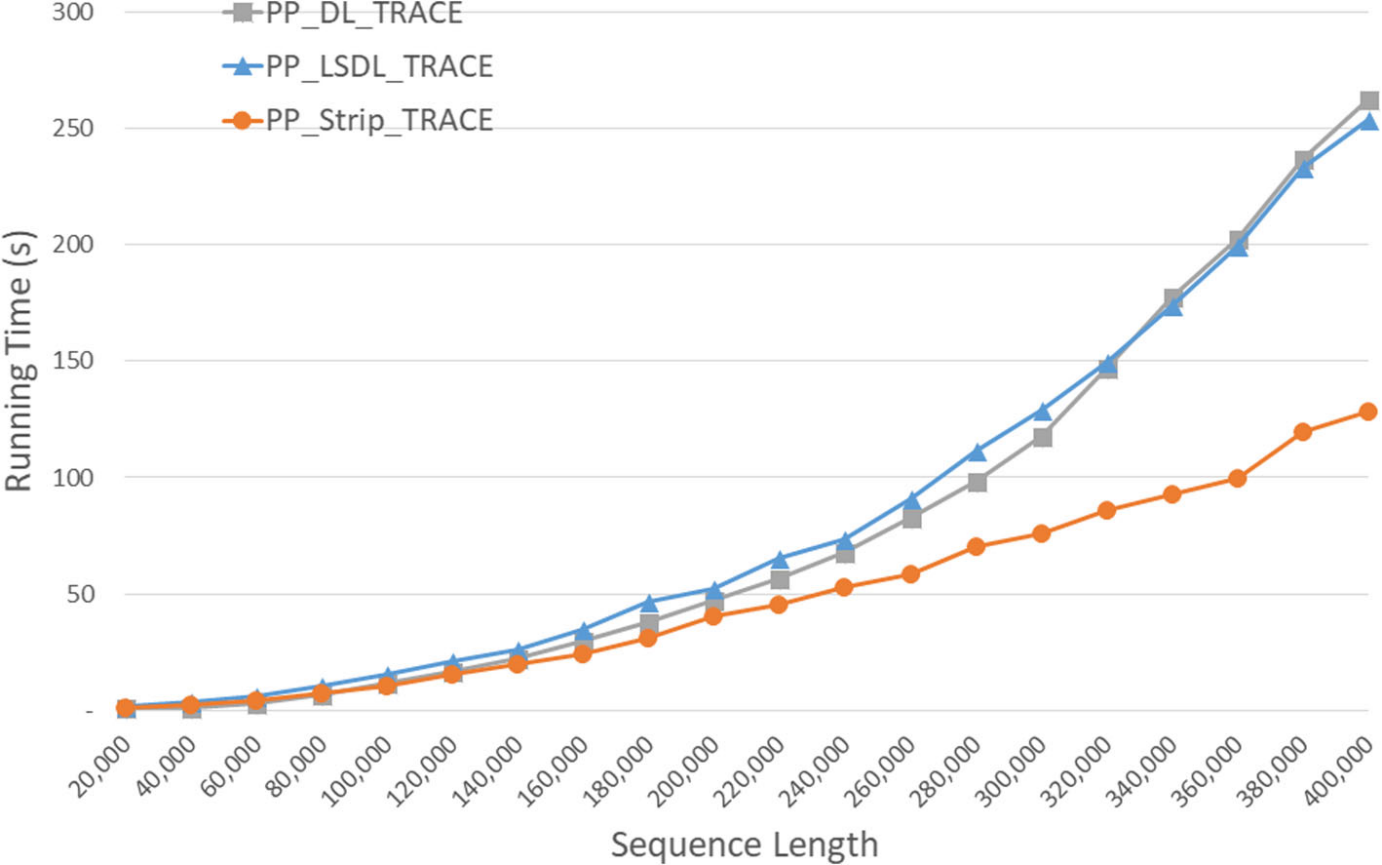
Run time of parallel DL trace algorithms, in seconds, on Xeon24

## Discussion

Cache efficient and multi-core linear-space algorithms to compute the DL distance between two strings as well as to determine an optimal trace (edit sequence) have been developed. The reduction in space provided by these algorithms enables the solution of much larger instances than is possible using previously known algorithms.

## Conclusion

Our algorithms were empirically evaluated on 3 computational platforms. Cache-misses were experimentally measured on one of these platforms and we verified that the algorithms analyzed to have a smaller number of cache misses using our simple cache model actually had fewer misses on a real computational platform. Significant run-time improvement (relative to known algorithms) was seen for our cache-efficient algorithms on all three platforms. On all platforms, the linear-space cache-efficient algorithms *Strip*_*DL* and *Strip*_*TRACE* were the best-performing single-core algorithms to determine the DL distance and optimal trace, respectively.

*Strip*_*DL* reduced run time by as much as 73.1*%* relative to the classical distance algorithm *DL* and *Strip*_*TRACE* reduced run time by as much as 63.5*%* relative to the classical trace algorithm. Multi-core versions of these two algorithms scaled quite well and achieved a speedup of up to 23.22 on a 24 core computer.

We also measured the energy efficiency of our algorithms on one of the platforms. Our best single-core algorithms reduced energy consumption by as much as 68.5% (relative to the best previously known algorithm) when computing the DL distance and by as much as 46.8% when computing an optimal trace. Our best multi-core algorithms achieves up to 81.4% and 57.6% energy consumption reduction, respectively.

## Abbreviations

CPU: Central processing unit
DL: Damerau-Levenshtein
DNA: DeoxyriboNucleic acid
LLC: Last level cache
LRU: Least recently used
NCBI: National center for biotechnology information
PDB: Protein data bank
RNA: RiboNucleic acid

## References

[1] Levenshtein VI (1966). Binary codes capable of correcting deletions, insertions, and reversals. Sov Phys Dokl.

[2] Damerau FJ (1964). A technique for computer detection and correction of spelling errors. Commun ACM.

[3] Maier D (1978). The complexity of some problems on subsequences and supersequences. J ACM.

[4] Robinson DJS (2003). An Introduction to Abstract Algebra.

[5] Jaro MA (1989). Advances in record-linkage methodology as applied to matching the 1985 census of Tampa, Florida. J Am Stat Assoc.

[6] Needleman SB, Wunsch CD (1970). A general method applicable to the search for similarities in the amino acid sequence of two proteins. J Mol Biol.

[7] Smith TF, Waterman MS (1981). Identification of common molecular subsequences. J Mol Biol.

[8] Lowrance R, Wagner RA (1975). An extension of the string-to-string correction problem. J ACM.

[9] Brill E, Moore RC (2000). An improved error model for noisy channel spelling correction. Proceedings of the 38th Annual Meeting on Association for Computational Linguistics.

[10] Bard GV (2007). Spelling-error tolerant, order-independent pass-phrases via the Damerau-Levenshtein string-edit distance metric. Proceedings of the Fifth Australasian Symposium on ACSW Frontiers - Volume 68. ACSW ’07.

[11] Li M, Zhang Y, Zhu M, Zhou M (2006). Exploring distributional similarity based models for query spelling correction. Proceedings of the 21st International Conference on Computational Linguistics and the 44th Annual Meeting of the Association for Computational Linguistics. ACL-44.

[12] Faloutsos C, Megalooikonomou V (2007). On data mining, compression, and kolmogorov complexity. Data Min Knowl Disc.

[13] Cai X, Zhang XC, Joshi B, Johnson R (2012). Touching from a distance: Website fingerprinting attacks and defenses. Proceedings of the 2012 ACM Conference on Computer and Communications Security.

[14] Majorek KA, Dunin-Horkawicz S, Steczkiewicz K, Muszewska A, Nowotny M, Ginalski K, Bujnicki JM (2014). The RNase H-like superfamily: new members, comparative structural analysis and evolutionary classification. Nucleic Acids Research, vol. 2.

[15] Protein Data Bank. http://www.rcsb.org/pdb/home/home.do. Accessed 15 Aug 2017.

[16] Hyyrö H (2003). A bit-vector algorithm for computing levenshtein and damerau edit distances. Nord J Comput.

[17] Oommen BJ, Loke R (1995). Pattern recognition of strings containing traditional and generalized transposition errors. 1995 IEEE International Conference on Systems, Man and Cybernetics. Intelligent Systems for the 21st Century, vol. 2.

[18] Damerau–Levenshtein Distance. https://en.wikipedia.org/wiki/Damerau-Levenshtein_distance. Accessed 15 Aug 2017.

[19] Zhao C, Sahni S (2015). Cache and energy efficient alignment of very long sequences. 5th IEEE International Conference on Computational Advances in Bio and Medical Sciences.

[20] Wagner RA, Fischer MJ (1974). The string-to-string correction problem. J ACM.

[21] Hirschberg DS (1975). A linear space algorithm for computing longest common subsequences. Commun ACM.

[22] Myers EW, Miller W (1988). Optimal alignments in linear space. Bioinformatics.

[23] Source Code Download Link. http://www.cise.ufl.edu/%7esahni/papers/DLDistanceAlgs.tar.gz. Accessed 15 Aug 2017.

[24] Perf Tool. https://perf.wiki.kernel.org/index.php/Main_Page. Accessed 15 Aug 2017.

[25] NCBI Database. http://www.ncbi.nlm.nih.gov/gquery. Accessed 15 Aug 2017.

